# Analysis of multiple-period group randomized trials: random coefficients model or repeated measures ANOVA?

**DOI:** 10.1186/s13063-022-06917-2

**Published:** 2022-12-07

**Authors:** Jonathan C. Moyer, Patrick J. Heagerty, David M. Murray

**Affiliations:** 1 Office of Disease Prevention, National Institutes of Health, Bethesda, MD USA; 2 Department of Biostatistics, University of Washington, Seattle, WA USA

**Keywords:** Cluster randomized trials, Group randomized trials, Multiple-period, Repeated measures, Random coefficients

## Abstract

**Background:**

Multiple-period parallel group randomized trials (GRTs) analyzed with linear mixed models can represent time in mean models as continuous or categorical. If time is continuous, random effects are traditionally group- and member-level deviations from condition-specific slopes and intercepts and are referred to as random coefficients (RC) analytic models. If time is categorical, random effects are traditionally group- and member-level deviations from time-specific condition means and are referred to as repeated measures ANOVA (RM-ANOVA) analytic models. Longstanding guidance recommends the use of RC over RM-ANOVA for parallel GRTs with more than two periods because RC exhibited nominal type I error rates for both time parameterizations while RM-ANOVA exhibited inflated type I error rates when applied to data generated using the RC model. However, this recommendation was developed assuming a variance components covariance matrix for the RM-ANOVA, using only cross-sectional data, and explicitly modeling time × group variation. Left unanswered were how well RM-ANOVA with an unstructured covariance would perform on data generated according to the RC mechanism, if similar patterns would be observed in cohort data, and the impact of not modeling time × group variation if such variation was present in the data-generating model.

**Methods:**

Continuous outcomes for cohort and cross-sectional parallel GRT data were simulated according to RM-ANOVA and RC mechanisms at five total time periods. All simulations assumed time × group variation. We varied the number of groups, group size, and intra-cluster correlation. Analytic models using RC, RM-ANOVA, RM-ANOVA with unstructured covariance, and a Saturated random effects structure were applied to the data. All analytic models specified time × group random effects. The analytic models were then reapplied without specifying random effects for time × group.

**Results:**

Results indicated the RC and saturated analytic models maintained the nominal type I error rate in all data sets, RM-ANOVA with an unstructured covariance did not avoid type I error rate inflation when applied to cohort RC data, and analytic models omitting time-varying group random effects when such variation exists in the data were prone to substantial type I error inflation unless the residual error variance is high relative to the time × group variance.

**Conclusion:**

The time × group RC and saturated analytic models are recommended as the default for multiple period parallel GRTs.

**Supplementary Information:**

The online version contains supplementary material available at 10.1186/s13063-022-06917-2.

## Background

Group- or cluster-randomized trials (GRTs) randomly assign groups or clusters to treatment conditions and measure members of those groups to assess the effect of an intervention. This may be done because the intervention is implemented at the group level, manipulates the physical or social environment, or simply cannot be delivered to individuals without substantial risk of contamination [1]. A key feature of such trials is that outcome measures within groups tend to be positively correlated—failing to account for this intraclass correlation coefficient (ICC) in trial design and analysis can result in inflated Type I error rates [1–5].

Multiple-period GRTs span two or more time periods, allowing for several design configurations [6]. Parallel GRTs randomly assign half of the groups to the intervention and follow all groups over time. Such designs can be modified to include one or more baseline periods in which no groups receive the intervention. These are examples of nested designs because each group appears in only one study condition. Cross-over designs, in which groups switch treatment status at least once over the course of the trial, are another type of multiple-period GRT [7, 8]. Stepped-wedge group-randomized trials are a form of one-way cross-over trial in which the intervention condition is implemented in groups on a staggered schedule until all groups receive the intervention [9]. These cross-over designs are examples of crossed designs because each group appears in each study condition. This paper is focused on analytic methods for parallel GRTs, which are always nested.

Multiple-period parallel GRTs can also be classified according to the presence of repeated observations. Designs in which the same individuals are measured at each time period are referred to as cohort designs, while designs in which individuals are measured only once are referred to as cross-sectional designs [1, 10, 11]. An example of a cross-sectional design with repeated measures on groups can be found in the Minnesota Heart Health Program [12]. In this study, six communities with 300–500 new individuals per community were surveyed at regular time periods on various health outcomes. In addition, the Minnesota Heart Health Program also included a cohort design with repeated measures on both groups and their members. Another example of a cohort parallel GRT is the Teens Eating and Nutrition Study, in which 16 schools with 52–344 students per school were followed over time to assess an intervention to improve nutrition among 7th and 8th graders [13]. In a review of parallel GRTs with cancer-related outcomes, Murray et al. [14] reported that 76.4% of those studies included a cohort design and 21.1% included a cross-sectional design while 2.1% included both; they also reported that 17.1% included two periods and another 6.5% included three or more periods, indicating parallel GRTs with repeated measures on groups and members are fairly common. Repeated observations on groups and members further complicates the correlation structure of outcome measures by introducing correlations across time at both the group- and member-level [1, 15, 16].

Analytic models for multiple-period parallel GRTs require several decisions about how best to specify the primary analysis. With this design, both individuals and clusters may be followed longitudinally and each warrant appropriate longitudinal correlation models. Key considerations include the representation of time in the mean model as categorical or continuous, the specification of random effects to generate both longitudinal correlations within an individual and correlation among individuals from the same cluster, choice of a covariance matrix for any random effects, and the degrees of freedom used for hypothesis tests of the intervention effect.

We begin with two classical strategies used for standard longitudinal analysis. First, traditional linear mixed models typically represent time as a continuous variable in group-specific mean models (with potential use of polynomial terms) and adopt random coefficients to induce correlation among repeated observations that share the same member- or cluster-specific trajectory. We refer to these models as random coefficient (RC) models, in which case random effects represent group- or member-level deviations from treatment condition specific intercepts and slopes. In contrast, repeated measures ANOVA models (RM-ANOVA) traditionally model time categorically using time-specific indicators and then adopt random effects that represent group- or member-level deviations from time-specific treatment group means. For standard longitudinal data analysis, RM-ANOVA models can adopt a simple random intercept model to induce covariance among repeated observations or can adopt more general random effects structures such as an exchangeable model for time-varying random effects. More generally, it is possible to assume a saturated or unstructured covariance matrix for repeated outcomes on an individual. Ultimately, longitudinal GRTs potentially require consideration of random effects at both the member-level and the cluster-level to characterize within-member correlation (for cohort designs) and within-cluster correlations. In generalizing RM-ANOVA models to GRTs, we refer to the general class of models specifying both time-invariant random effects (at the member or group level) and time-varying random effects (at the member or group level) as RM-ANOVA models, while models specifying only time-varying random effects at both levels are referred to as Saturated models.

Relatively few existing works have compared RM-ANOVA and RC approaches in the context of the design and analysis of parallel GRTs. Motivated by the design challenges of the Minnesota Heart Health Program [12], Murray et al. [17] explored the performance of RC models with UN covariance matrix and RM-ANOVA with VC covariance matrix on cross-sectional, parallel GRT data generated assuming RC or RM-ANOVA mechanisms. They observed that RC analytic models maintained nominal type I error rates for both data generation mechanisms; however, RM-ANOVA analytic models showed inflated type I error rates when applied to RC data. As it is common to use information criteria to select models, Murray et al. [17] also identified AIC and BIC favored models for their simulated data sets and found that these models often exhibited an inflated type I error rate. Thus, RC analytic models were recommended for multiple-period parallel GRT data and use of AIC or BIC to select a different model was discouraged. More recently, Kasza and Forbes [18] investigated the impact of mis-specifying correlation structures in the RM-ANOVA setting—for example, assuming correlation between outcomes within the same group at different time periods is the same when in fact the correlation decays as a function of time. As their primary focus was studying misspecification of decaying correlation structure, they did not investigate RC analytic models but noted that further work in this area is required.

Correct specification of the random effects structure is important for obtaining proper estimates of parameters and their standard errors [19]. In the context of linear mixed models, Bell et al. [20] show that omitting random slopes in analytic models when such variation exists in the data generating mechanism results in standard error estimates that are too small. Bell and Rabe [21] applied the mixed model for repeated measures frequently used in longitudinal individually randomized trials to multiple-period parallel GRTs. In the terminology defined above, this analytic model is an RM-ANOVA analytic model for cohort data with a random effect for group, no time × group random effect, and an UN covariance matrix at the member level and a VC covariance matrix at the group level. They found the model maintained nominal type I error rate across a range of ICCs when applied to data generated assuming an RM-ANOVA mechanism with no time × group component of variation. In contrast, if the data generation mechanism included a time × group component of variation but the analytic model did not, the type I error rate was inflated, with the level of inflation increasing as the magnitude of the time × group component of variation increased. Importantly, Bell and Rabe [21] did not evaluate their RM-ANOVA model for data generated assuming a RC model.

In the context of RM-ANOVA and RC analytical models, three primary questions guide this work. First, would Murray et al. [17] have found better performance with RM-ANOVA models using an unstructured covariance matrix? In their conclusions, Bell and Rabe [21] noted their model was not explored by Murray et al. [17] and that the recommendations of the latter were therefore “too broad.” This observation prompted investigation of the UN covariance matrix in this work. Second, would Murray et al. [17] have seen patterns in cohort data similar to those they saw with cross-sectional data? Such data requires specification of a more complicated covariance structure than in cross-sectional data. Third, how important is the time × group random effect term in the analytic model if the data generation mechanism also includes variability at that level? Murray et al. [17] did not explore the ramifications of omitting the time × group random effect in RM-ANOVA and RC analytic models if the data generation mechanism includes variability at that level. While inflated type I error rates may be expected for the RM-ANOVA model based on Bell and Rabe [21], the performance of RM-ANOVA analytic models compared to RC analytic models in this regard is not known.

In this work, we expand on the Monte Carlo analysis of type I error rate for the hypothesis of no fixed effect interaction in Murray et al. [17] and Bell and Rabe [21] to address these questions. In the “Background” section, we provide background related to these issues and present RM-ANOVA and RC models. The “Method” section details the simulation procedures and methods used to address the questions of interest. Results are presented in the “Results” section, with further discussion in the “Discussion” section. In the “Conclusions” section, we summarize our finding and present conclusions.

## Method

### Data generation

In this section, we present the repeated measures ANOVA (RM-ANOVA) and random coefficients (RC) data generation mechanisms. Next we discuss the various correlations important to characterizing within- and between-group variation, in the same time period or across time. Finally, we present details on data generation parameters.

#### Repeated measures ANOVA (RM-ANOVA) model

We first consider a nested cross-sectional multiple-period parallel GRT design, where individuals are measured only once in each time period. Let $Y_{ijkl}$ be a continuous outcome for the $i$th member ($i = 1, \dots , m$) nested within the $k$th group ($k = 1, \dots , g$) and the $l$th condition ($l = 1, \dots , c$) at time $j$ ($j = 1, \dots , t$). The cross-sectional RM-ANOVA model is as follows:

1 $$\begin{aligned} Y_{ijkl} = \mu + C_l + T_j + TC_{jl} + G_{kl} + TG_{jkl} + \epsilon _{ijkl} \end{aligned}$$

where $\mu$ is the mean outcome in the control condition at baseline, $C_l$ the baseline difference between the mean of the $l$th condition and control condition mean ($C_1=0$), $T_j$ is the difference between the mean outcome of the $j$th time period with baseline mean in the control condition ($T_1=0$), $TC_{jl}$ is the time by condition interaction for the $l$th condition at the jth time period ($TC_{1l}=TC_{j1}=0$), $G_{kl}$ is a random intercept for the $k$th group in the $l$th condition, $TG_{jkl}$ is a random intercept for group $k$ in condition $l$ at time $j$, and $\epsilon _{ijkl}$ is random member-level measurement error. Random effects are assumed to be independent and distributed as $G_{kl} \sim N(0,\sigma _{g}^2)$, $TG_{jkl} \sim N(0,\sigma _{tg}^2)$, and $\epsilon _{ijkl} \sim N(0, \sigma _e^2)$.

To account for the cohort data structure, Eq. 1 can be extended by adding member-level random effects as follows:

2 $$\begin{aligned} Y_{ijkl} = \mu + C_l + T_j + TC_{jl} + G_{kl} + TG_{jkl} + M_{ikl} + TM_{ijkl} + \epsilon _{ijkl} \end{aligned}$$

where $M_{ikl}$ is the random intercept for the $i$th member of the $k$th group in the $l$th condition and $TM_{ijkl}$ is the random intercept for the $i$th member of the $k$th group in the $l$th condition at the $j$th time period. These random effects are assumed to be independent of all other random effects and distributed as $M_{ikl} \sim N(0,\sigma _{m}^2)$ and $TM_{ijkl} \sim N(0,\sigma _{tm}^2)$.

As is common with most trials, in this work, we assume only one observation per member per measurement occasion. In such a setting, $TM_{ijkl}$ cannot be distinguished from residual error and will be omitted from model 2. However, if members have multiple observations per measurement occasion, then it is possible to separately estimate the variance for the time × member random effect $TM_{ijkl}$ and the residual error variance.

#### Random coefficients (RC) model

The RC model represents time as a continuous variable and further considers a random slope for the continuous time variable. For cross-sectional data, the RC model is given by the following:

3 $$\begin{aligned} Y_{ijkl} = \mu + C_l + T_{\text {(lin)}}t_j + T_{\text {(lin)}}C_{l}t_j + G_{kl} + T_{\text {(lin)}}G_{kl}t_j + \epsilon _{ijkl} \end{aligned}$$

where $T_{\text {(lin)}}$ is the linear time slope in the control condition, $t_j$ is the value of time at the $j$th period, $T_{\text {(lin)}}C_{l}$ is the interaction between the $l$th condition and $j$th time point ($T_{(lin)}C_1=0$), and $T_{\text {(lin)}}G_{kl}$ is a random slope for the $k$th group in the $l$th condition such that $T_{\text {(lin)}}G_{kl} \sim N(0, \sigma _{t(\text {lin})g}^2)$, with other terms defined as for model 1. Random effects $G_{kl}$ and $T_{\text {(lin)}}G_{kl}$ are assumed to be independent of $\epsilon _{ijkl}$, but they need not be independent from each other.

Extending model 3 to accommodate the cohort design requires the addition of random effects for member-level intercepts and slopes as follows:

4 $$\begin{aligned} Y_{ijkl} = \mu + C_l + T_{\text {(lin)}}t_j + T_{\text {(lin)}}C_{l}t_j + G_{kl} + T_{\text {(lin)}}G_{kl}t_j + M_{ikl} + T_{(lin)}M_{ikl}t_j + \epsilon _{ijkl} \end{aligned}$$

where $T_{(lin)}M_{ikl}$ is the random slope for the $i$th member in the $kl$th group, with $T_{(lin)}M_{ikl} \sim N(0, \sigma _{t(\text {lin})m}^2)$. Similar to the group-level random effects $G_{kl}$ and $T_{\text {(lin)}}G_{kl}$, $M_{kl}$ and $T_{\text {(lin)}}M_{kl}$ are assumed to be independent of other random effects but may covary with each other. More details on the cohort RC model can be found elsewhere [16]. In contrast to RM-ANOVA, one observation per member per measurement occasion is sufficient to estimate the variance component for the time × member random effect $T_{(lin)}M_{ikl}$.

#### Within- and between-period intracluster correlations (ICC)

In single-period parallel GRTs, an important parameter is the intraclass correlation (ICC) which can be defined as the average bivariate correlation among observations taken in the same group or as the fraction of the total variation in the outcome attributable to groups. For multiple-period parallel GRTs, more complicated random-effects structure gives rise to within- and between-period correlations [6]. These quantities provide information on the similarity among outcome values due to correlation within groups or clusters and to repeated measures on the same groups or clusters or on the same members.

The within-period ICC (WPICC) is a measure of the similarity among values on the outcome variable for two different members of the same group or cluster within a given time period and is equivalent to the ICC in a single-period GRT. In the cross-sectional RM-ANOVA setting, the WPICC implied by model 1 can be calculated as

5 $$\begin{aligned} WPICC = \frac{\sigma _{g}^2 + \sigma _{tg}^2}{\sigma _{g}^2 + \sigma _{tg}^2 + \sigma _{e}^2} \end{aligned}$$

In the cohort RM-ANOVA setting, the WPICC implied by model 2 is defined as

6 $$\begin{aligned} WPICC = \frac{\sigma _{g}^2 + \sigma _{tg}^2}{\sigma _{g}^2 + \sigma _{tg}^2 + \sigma _{m}^2 + \sigma _{tm}^2 + \sigma _{e}^2} \end{aligned}$$

Between-period ICC (BPICC) measure similarity among values on the outcome variable across time. The BPICC implied by model 1 is

7 $$\begin{aligned} BPICC = \frac{\sigma _{g}^2}{\sigma _{g}^2 + \sigma _{tg}^2 + \sigma _{e}^2} \end{aligned}$$

and the BPICC implied by model 2 is

8 $$\begin{aligned} BPICC = \frac{\sigma _{g}^2}{\sigma _{g}^2 + \sigma _{tg}^2 + \sigma _{m}^2 + \sigma _{tm}^2 + \sigma _{e}^2} \end{aligned}$$

Between-period correlations are often expressed in terms of cluster autocorrelation (CAC) and individual autocorrelation (IAC) [6, 10, 14]. CAC is the correlation between the population means for the outcome from the same group or cluster at two different time periods and is present in both cross-sectional and cohort data. Sometimes called the over-time correlation at the group level [1], CAC is equivalent to the ratio of BPICC to WPICC.

9 $$\begin{aligned} CAC = \frac{BPICC}{WPICC} = \frac{\sigma _{g}^2}{\sigma _{g}^2 + \sigma _{tg}^2} \end{aligned}$$

Individual autocorrelation (IAC) is the correlation on the outcome variable for the same individual at two different time periods and is present only in cohort designs. The IAC is sometimes called the over-time correlation at the member level [1].

10 $$\begin{aligned} IAC = \frac{\sigma _{m}^2}{\sigma _{m}^2 + \sigma _{tm}^2 + \sigma _{e}^2} \end{aligned}$$

We make three remarks regarding these expressions. First, as mentioned previously, most trials measure individuals once per measurement occasion. Thus, the time × member random effect variance $\sigma _{tm}^2$ is indistinguishable from residual error variance and is typically omitted from these expressions in other sources. Second, recent work in multiple-period GRTs in the RM-ANOVA setting has focused on exponentially decaying cluster and individual autocorrelations over time [11, 22]. We do not consider such model extensions in this work, but it should be noted that misspecifying the exponentially decaying structure can have a strong impact on the type I error rate for testing treatment effect. Finally, in the RM-ANOVA setting, BPICC is constant over time while in the RC setting BPICC is a non-constant function of time; the explicit expression is given in [23]. For ease of presentation, in this work, we represent all WPICCs using the RM-ANOVA definitions given by Eqs. 5 and 6.

#### Data generation parameter settings

To address the three research questions indicated in “Background” section, data sets were repeatedly simulated for cross-sectional and cohort multiple-period GRTs for both RM-ANOVA and RC models given in Eqs. 1–4. Note that these models all contained time × group variation, so all simulated data were generated assuming variability at that level. All random effects were assumed to be independent. This includes the effects $G_{kl}$ and $T_{\text {(lin)}}G_{kl}$ in both models 3 and 4, as well as random effects $M_{kl}$ and $T_{\text {(lin)}}M_{kl}$ random effects in model 4.

All simulations assumed two conditions, five equally spaced time periods (0 to 4), and 40 members per group. The number of groups per condition varied over 10, 20, and 40. All fixed effects parameters were set to 0. The variances of the group and time × group random effects were set to 1, resulting in a CAC of 0.50. Other variance values were chosen to obtain within-period ICCs in the RM-ANOVA framework of 0.10, 0.01, and 0.001 and an IAC of 0.70 for cohort models. This value of IAC was chosen because member-level effects are not expected to impact analysis unless the individual autocorrelation is large [24, 25]. While there is no one threshold for what constitutes a large IAC, we use a value similar to Bell and Rabe [21]. For cross-sectional models 1 and 3, residual error variances were set to 18, 198, and 1998. For cohort models 2 and 4, member-level and residual error variances were set using the values listed in Table 1. For each combination of the two data structures (cross-sectional and cohort), three values for groups per condition, and three ICCs, we simulated 1000 data sets.

**Table 1:** Table of variance values for member random effects, time by member random effects, and residual error for RM-ANOVA and RC cohort data generating mechanisms. The variance values were chosen to have an individual autocorrelation of 0.70

Data generation mechanism	$\sigma _m^2$	$\sigma _{t(\text {lin})m}^2$	$\sigma _e^2$	ICC
RM-ANOVA	12.6		5.4	0.10
	12.6		59.4	0.01
	12.6		599.4	0.001
RC	12.6	1	4.4	0.10
	12.6	11	48.4	0.01
	12.6	111	488.4	0.001

### Analytic models

Several analytic models were fit to the simulated data sets using the PROC MIXED procedure of SAS software, Version 9.4 of the SAS System for Windows. Before providing details on these analytic models, we review the generic formulation of a general linear mixed model in the context of a longitudinal study to explain different constraints on covariance matrices. This facilitates the subsequent discussions on specific models for longitudinal GRTs.

#### Covariance structures

The general linear mixed model for the outcome from the ith member is given as follows:

11 $$\begin{aligned} {\textbf {Y}}_i = {\textbf {X}}_i\beta + {\textbf {Z}}_i \gamma _i + \epsilon _i \end{aligned}$$

where ${\textbf {Y}}_i$ is the vector of repeated outcome measures, ${\textbf {X}}_i$ is the fixed effects design matrix, $\beta$ is the vector of fixed effects, ${\textbf {Z}}_i$ is the random effects design matrix, $\gamma _i$ is the subject-specific vector of random effects, and $\epsilon _i$ is residual error. We assume vectors $\gamma _i$ and $\epsilon _i$ are independent and $\gamma _i \sim N(0, \textbf{G})$ and $\epsilon _i \sim N(0, \textbf{R})$, where $\textbf{G}$ and $\textbf{R}$ are the between- and within-subject random effects covariance matrices, respectively.

SAS PROC MIXED offers several options to fit covariance matrices $\textbf{G}$ and $\textbf{R}$ using the TYPE option in the RANDOM and REPEATED statements, respectively. One structure is variance components (VC), which models an independent variance component for each random term. For example, fitting a VC structure to model 3 with five time periods yields the following $\textbf{G}$ matrix in SAS:

$$\begin{aligned} \left[ \begin{array}{cccccc} \sigma _{g}^2 &{} 0 &{} 0 &{} 0 &{} 0 &{} 0 \\ 0 &{} \sigma _{tg}^2 &{} 0 &{} 0 &{} 0 &{} 0 \\ 0 &{} 0 &{} \sigma _{tg}^2 &{} 0 &{} 0 &{} 0 \\ 0 &{} 0 &{} 0 &{} \sigma _{tg}^2 &{} 0 &{} 0 \\ 0 &{} 0 &{} 0 &{} 0 &{} \sigma _{tg}^2 &{} 0\\ 0 &{} 0 &{} 0 &{} 0 &{} 0 &{} \sigma _{tg}^2 \\ \end{array}\right] \end{aligned}$$

The first entry in the diagonal represents the variance of random effect $G_{kl}$, while the remaining entries correspond to the five $TG_{jkl}$ random effects for each of the five time periods.

Another approach is to use a compound symmetric (CS) structure, which assumes random effects have the same variance at each time period and a constant covariance. For example, the $R$ matrix of model 2 could be given by:

$$\begin{aligned} \left[ \begin{array}{ccccc} \sigma _{m}^2 + \sigma _{e}^2 &{} \sigma _{m}^2 &{} \sigma _{m}^2 &{} \sigma _{m}^2 &{} \sigma _{m}^2 \\ \sigma _{m}^2 &{} \sigma _{m}^2 + \sigma _{e}^2 &{} \sigma _{m}^2 &{} \sigma _{m}^2 &{} \sigma _{m}^2 \\ \sigma _{m}^2 &{} \sigma _{m}^2 &{} \sigma _{m}^2 + \sigma _{e}^2 &{} \sigma _{m}^2 &{} \sigma _{m}^2 \\ \sigma _{m}^2 &{} \sigma _{m}^2 &{} \sigma _{m}^2 &{} \sigma _{m}^2 + \sigma _{e}^2 &{} \sigma _{m}^2 \\ \sigma _{m}^2 &{} \sigma _{m}^2 &{} \sigma _{m}^2 &{} \sigma _{m}^2 &{} \sigma _{m}^2 + \sigma _{e}^2 \\ \end{array}\right] \end{aligned}$$

Diagonal elements of the matrix denote member-level variation within a time period, while off-diagonal elements represent the covariance between member-level outcomes across time periods.

Finally, an unstructured (UN) covariance matrix allowing variances for each random effect as well as the covariances between them may be used. For example,the $R$ matrix of model 2 could be fitted as

$$\begin{aligned} \left[ \begin{array}{ccccc} \sigma _{1}^2 &{} \sigma _{12} &{} \sigma _{13} &{} \sigma _{14} &{} \sigma _{15} \\ \sigma _{12} &{} \sigma _{2}^2 &{} \sigma _{23} &{} \sigma _{24} &{} \sigma _{25} \\ \sigma _{13} &{} \sigma _{23} &{} \sigma _{3}^2 &{} \sigma _{34} &{} \sigma _{35} \\ \sigma _{14} &{} \sigma _{24} &{} \sigma _{34} &{} \sigma _{4}^2 &{} \sigma _{45} \\ \sigma _{15} &{} \sigma _{25} &{} \sigma _{35} &{} \sigma _{45} &{} \sigma _{5}^2 \\ \end{array}\right] \end{aligned}$$

where $\sigma _{j}^2$ denotes the member-level variation at time $j$ and $\sigma _{jj^{\prime }}$ denotes the covariance between member-level observations at times $j$ and $j^{\prime }$. SAS offers a variation of UN covariance called UN(1), which is an unstructured matrix with off-diagonal elements equal to 0.

The choice of covariance structure comes with benefits and drawbacks [26]. VC and CS require estimation of relatively few variance parameters, but they are simple structures and may not adequately characterize the variance structure in a real data set. Conversely, UN covariance requires estimating substantially more parameters, but is flexible and can be more widely applicable to any data set with an adequate sample size.

#### Analytic models

Four analytic models were fit to each replication. All models possessed fixed effects for time, condition, and their interaction.

The first analytic model—“RM-ANOVA with VC covariance”—specified random effects for group and and time × group. The variance components (VC) covariance structure for the $\textbf{G}$ matrix was used when the model was applied to both cross-sectional and cohort data sets. For cohort data sets, a compound symmetric $\textbf{R}$ matrix was also used. The cross-sectional version of RM-ANOVA with VC covariance is equivalent to the RM-ANOVA data generation mechanism shown in model 1. While the cohort version of the RM-ANOVA with VC covariance analytic model did not directly specify a member-level random effect, with the use of a compound symmetric $\textbf{R}$ matrix this analytic model is equivalent to the data generation mechanism shown in model 2.

The second analytic model—“RM-ANOVA with UN covariance”—specified random effects for group and time × group. For cross-sectional data, an unstructured $\textbf{G}$ matrix was specified. For cohort data, variance components and unstructured covariance structures were used for the $\textbf{G}$ and $\textbf{R}$ matrices, respectively.

The third analytic model—“RC”—specified random effects for group and time × group. An unstructured covariance structure for the $\textbf{G}$ matrix for both cross-sectional and cohort data was used. For cohort data, RC analytic models also specified random effects for member and time × member. For both cross-sectional and cohort data, an unstructured covariance structure was used for the $\textbf{G}$ matrix. The cross-sectional and cohort versions of the RC analytic model are equivalent to the RC data generation mechanisms given by models 3 and 4, respectively.

The fourth analytic model—“Saturated”—specified an unstructured $\textbf{G}$ matrix for both cross-sectional and cohort dataset. Cohort Saturated models also utilized an unstructured $\textbf{R}$ matrix, while cross-sectional Saturated models employed an unstructured $\textbf{R}$ matrix with off-diagonal elements equal to 0, i.e., “type” option of UN(1). Notably, Saturated models did not specify time-invariant random effects for group as the preceding three analytic models did.“Saturated” was chosen as a name because a consequence of using unstructured covariance matrices and time-varying random effects is that as many within- and between-period variance-covariance parameters are estimated as is possible.

Table 2 summarizes the $\textbf{G}$ and $\textbf{R}$ matrices specifications for these analytic models, along with the number of covariance parameters estimated for models fit to individual-level data and—where indicated—mean models. Analytic model code is provided in Supplemental material. As these four analytic models possessed time-varying group random effects, they are referred to as “time × group” models.

**Table 2:** Table of analytic models and specifications for $\textbf{G}$ and $\textbf{R}$ matrices, along with the number of covariance parameters. A hyphen in the R matrix columns indicates that no R-matrix type was specified. For the parameters, numbers appearing to the left of the comma pertain to analytic models containing time × group random effects, while numbers to the right pertain to analytic models containing only group-intercept random effects. Values appearing in parentheses correspond to to the number of covariance parameters for the mean model. Note that Saturated analytic models did not fit time-invariant group random effects

	Cross-sectional			Cohort		
Model	G Matrix	R Matrix	Parameters	G Matrix	R Matrix	Parameters
RM-ANOVA with VC	VC	-	3, 2	VC	CS	4, 3
RM-ANOVA with UN	UN	-	16 (15), 2	VC	UN	17, 16
RC	UN	-	4, 2	UN	-	7, 4
Saturated	UN	UN(1)	20 (15), -	UN	UN	30 (15), -

Recall that all data were generated with variation at the time × group level. To assess the impact of omitting time × group random effects in the analysis, we also fit versions of the RM-ANOVA and RC analytic models omitting the time × group random effect. Note that this could not be done for the saturated model as it contains only time × group random effects. Analytic models omitting time × group random effects are referred as “intercept only” models. The model utilized by Bell and Rabe [21] corresponds to the intercept only RM-ANOVA, UN for cohort data.

Most analytic models were fit on individual-level data. Exceptions included the cross-sectional, time × group RM-ANOVA with UN covariance analytic models as well as both cross-sectional and cohort saturated analytic models. In these cases, the analysis was conducted on the group means. When group sizes are the same, the group mean model formulation is not expected to be much less efficient than the individual level formulation [5], but the former can dramatically reduce model fitting time compared to the latter. This was borne out in test simulations, in which F-test results for the the intervention effect yielded the same *p*-values. A consequence of this is that the results for cross-sectional time × group RM-ANOVA with UN covariance are identical to cross-sectional saturated analytic models. This makes sense, as these two analytic models just apportion the total variance found in the denominator of Eq. 5 differently.

To assess the performance of the various analytic models, type I error rates were estimated under the null hypothesis of no fixed effect time × condition interaction—$TC_{jl}$ for RM-ANOVA and Saturated analytic models and $T_{\text {(lin)}}C_{l}$ for RC. For RM-ANOVA and saturated analytic models, the null hypothesis is that there is no difference in the pattern of condition means over time between condition $l$ and the control condition, while for the RC analytic model the null hypothesis is that there is no difference in linear slope between intervention condition $l$ and the control condition. The “nobound” option was used with all models to remove the non-negativity constraint when estimating variance components, which has been shown to maintain nominal type I error rates [27]. In addition, all models were fit using restricted maximum likelihood. The nominal level of significance for all tests was specified at 0.05. Kenward-Rogers degrees of freedom were specified in the “ddfm” option - specifically, “kr2” [28, 29]. This setting has been shown to give good performance relative to other denominator degrees of freedom estimation methods across a range of settings common to GRTs [30, 31]. A drawback when conducting simulations using Kenward-Roger degrees of freedom is that the approach tends to require more computing resources and therefore may take an extended period of time to run. The use of Kenward-Roger degrees of freedom is a change from the earlier work of Murray et al. [17], which employed BW degrees of freedom. We conducted a round of simulations using BW degrees of freedom and include these results as Supplementary data. The proportion of the 1000 replications with *p*-values below the level of significance provided an estimate of the type I error rate of the analytic model in question. The ggplot2 R package was used to generate plots of estimated type I error rate as a function of the WPICC assuming RM-ANOVA as shown in Eqs. 5 and 6 [32]. The kableExtra R package [33] was used to generate LaTeX tables displaying type I error rates for the various parameter settings, which are added as Supplementary data.

## Results

**Fig. 1:**
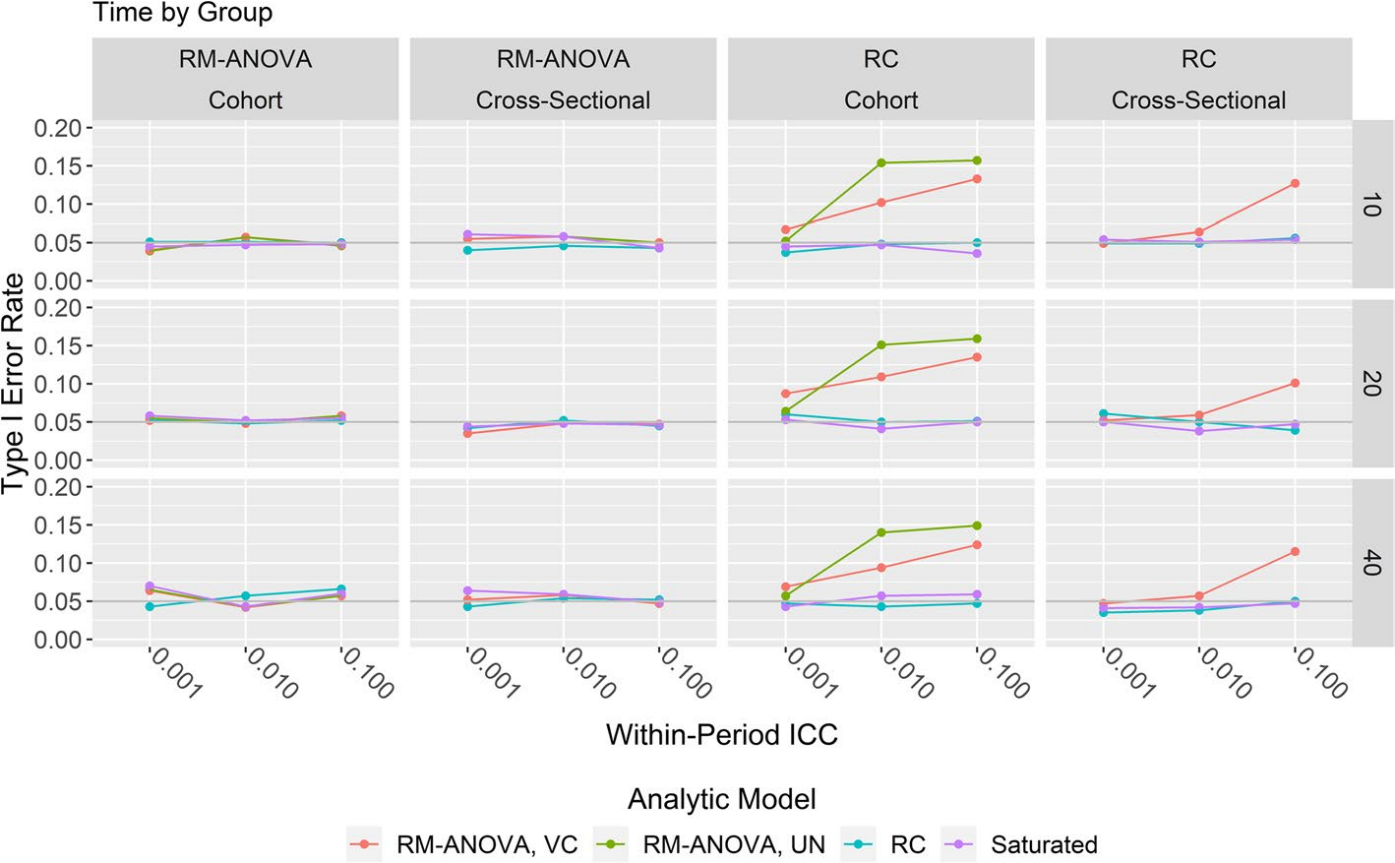
Time by group analytic model results

**Fig. 2:**
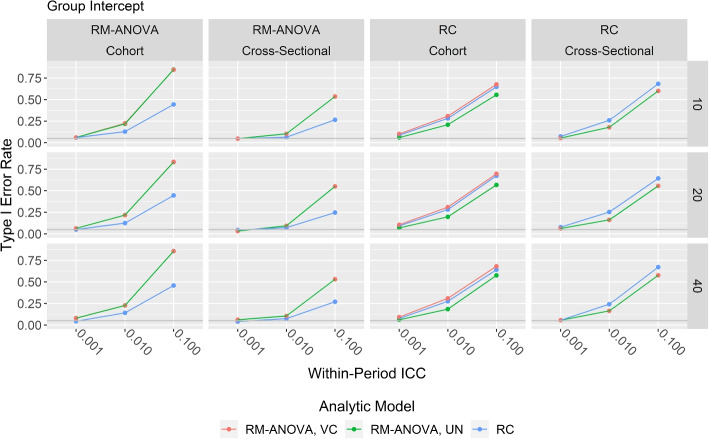
Intercept only analytic model results

Simulation results for applying the various analytic models to data generated according to models 1 to 4 are given in Figs. 1 and 2. For each panel, the *y*-axis is the estimated type I error rate and the *x*-axis is the within-period ICC in descending order. Each column of panels corresponds to a data generation mechanism, while each row of panels corresponds to the number of groups per condition. Results of the analytic models are indicated by line color in each panel. The gray horizontal line near the bottom of each panel corresponds to the nominal type I error rate of 0.05.

Figure 1 shows results for analytic models specifying time × group random effects. As seen in the two left columns, all four analytic models performed well when applied to RM-ANOVA data. However, the two right columns indicate that the RM-ANOVA with VC covariance matrix struggled with RC data unless the ICC was low, which happens only when there is little variation attributable to both group and time × group. The RM-ANOVA with UN covariance matrix analytic model performed poorly on RC cohort unless the ICC was low. The RC and Saturated analytic models performed well in all situations.

Figure 2 shows results for the intercept only analytic models that do not account for time × group variation. Note that the saturated model is omitted as it contains only time-varying random effects, and that the scale of the *y*-axis is much wider than that of Fig. 1. For data generated with an RM-ANOVA mechanism, the RC analytic model performed best, but all three analytic models attained nominal type I error rates only when the ICC was at its lowest value. For data generated with an RC mechanism, the RM-ANOVA with UN covariance matrix performed slightly better than the other two analytic models at all values of the ICC. All three analytic models specifying only group-level intercepts performed poorly unless the ICC was very low.

Note that the type I error rates for both RM-ANOVA analytic models in Fig. 2 are the same when applied to cross-sectional data. This is to be expected, as the $\textbf{G}$ matrix in both models only contains one variance term corresponding to the group-level random intercept $G_{kl}$.

In our work, we set the variances of the group random effect $G_{kl}$ and time × group random effects $TG_{kl}$ and $T_{(lin)}G_{kl}$ for RM-ANOVA and RC data models, respectively, to 1. This yielded a cluster autocorrelation of 0.5. However, in practice the variance of the time × group random effect is often small with respect to that of the group random effect. To account for this, we repeated the simulation study with time × group random effect variance set to 0.10, yielding a cluster autocorrelation of 0.91. This modification did not change the overall pattern of results.

## Discussion

The goal of this work was threefold. The first goal was to assess the performance of RM-ANOVA analytic models with UN covariance relative to those with VC structure when applied to cross-sectional data. Both analytic models performed well when applied to cross-sectional RM-ANOVA data when using Kenward-Roger degrees of freedom. This setting is important, as using BW degrees of freedom resulted in RM-ANOVA with UN covariance having inflated type I error rates when the number of groups per arm was low. Both RM-ANOVA analytic models did well when applied to cross-sectional RC data as long as the ICC was low and Kenward-Roger degrees of freedom was used. However, if the ICC was large, RM-ANOVA with VC covariance exhibited inflated type I error rates. As noted in Murray et al. [17], RM-ANOVA analytic models with VC covariance exhibited inflated type I error rate when group-specific slopes were heterogeneous. RM-ANOVA with UN covariance can accommodate this heterogeneity of trends, but a key component is the use of Kenward-Roger degrees of freedom, which offers a more conservative estimate of degrees of freedom than the design-based BW approach. We note that RC and saturated analytic model exhibited nominal type I error rates when applied to cross-sectional data, with the use of Kenward-Roger degrees of freedom being important with the Saturated model.

The second goal was comparing the performance of RC and RM-ANOVA analytic models when applied to cohort data. The correctly specified RC analytic model performed well in terms of type I error control on cohort data generated assuming both mechanisms. Both RM-ANOVA analytic models performed well on cohort RM-ANOVA data when Kenward-Roger degrees of freedom were used, but not RC cohort data unless the ICC was low. Like the cohort time × group RC analytic model, the saturated model with Kenward-Roger degrees of freedom did well when applied to data generated assuming both mechanisms.

Finally, the importance of including time × group in the analytic model if the data contain variability at that level was investigated. As seen in Fig. 2, severe inflation of type I error rates was observed unless the ICC was small, which would happen only if the group and time × group components of variance were both small. This finding is consistent with the recent report from Bell and Rabe [21]. These patterns held for both Kenward-Roger and BW degrees of freedom. The necessity of including time × group random effect in the analytic model has also been emphasized in the existing literature for stepped wedge designs [23, 34].

In this study, we applied analytic models to data generated with both group and time × group random effects. During review, it was suggested we apply our analytic models to data generated with a group random effect but no time × group random effect. We did this for all cross-sectional data generation settings, but due to time constraints only generated cohort data having 10 and 20 groups per arm and nominal ICCs of 0.01 and 0.10. We found little impact on type I error rates if time × group random effects were included in the analytic model. This supports the general recommendation of including time × group random effects in the analysis of multiple period GRT data as there is little indication of any penalty of having it and the potential for a substantial problem if left out. This is consistent with other findings in the literature suggesting that the drawbacks of over-fitting tend to be less severe than those associated with under-fitting [35–37].

In summary, we offer the following recommendations regarding the time × group analytic models used in this work. RM-ANOVA with VC covariance exhibits type I error inflation when applied to RC data, with either BW or Kenward-Roger degrees of freedom. As the data generation mechanism is unknown, this suggests avoiding the use of RM-ANOVA with VC in general. Similarly, RM-ANOVA with UN covariance exhibits inflated type I error rates when applied to cross-sectional RC data with either BW or Kenward-Roger degrees of freedom. Thus, another conclusion is to avoid the use of RM-ANOVA with UN, which disagrees with Bell and Rabe [21]. RC analytic models performed well across all data sets, as did Saturated analytic models when used in conjunction with Kenward-Roger degrees of freedom. Indeed, the use of Kenward-Roger degrees of freedom can be generally recommended, as it offers benefits when used with UN covariance matrices with no price in type I error. Fitting analytic models on individual-level data with Kenward-Roger degrees of freedom can take a considerable amount of time when the number of groups per arm is large and the ICC is low, but with the saturated model it is straightforward and fast to apply the analytic model to the group means at each period.

We focused on VC and UN covariance structures for the $\textbf{G}$ matrix in our RM-ANOVA analytic models, but statistical software offers many other options such as compound symmetric or Toeplitz structures. As with the VC covariance structure, these covariance structures have fewer parameters to fit than the UN covariance structure, but they require adequate support in the data to justify. The UN covariance structure can be applied to all data sets, but this flexibility comes at the cost of fitting many parameters. In the context of RM-ANOVA analytic models with the UN covariance matrix, the number of covariance parameters increases dramatically as the number of time periods increases. Therefore, to maintain nominal type I error rates in these settings, it is important to employ a more conservative approach to estimating denominator degrees of freedom, such as the Kenward-Roger approach. RC analytic models have an advantage in this regard as they generally require estimating many fewer covariance parameters.

This work focused primarily on type I error rates in analytic models, but we say a few words about statistical power here. Our RC analytic model assumed a linear slope with time, which may result in reduced power if this assumption is violated. If this is a concern, the RC model can easily be extended to accommodate higher-order terms with time to improve power, provided enough groups per arm exist to provide degrees of freedom for the additional parameters. Alternatively, the Saturated model using Kenward-Roger degrees of freedom can be employed. While calculating power in this setting using simulation is straightforward, methods for calculating power and sample size assuming Kenward-Roger degrees of freedom have recently become available [38].

A common form of model selection is the use of information criteria such as AIC and BIC. This approach was explored in Murray et al. [17], who found that AIC- and BIC-favored models had inflated type I error rates in some situations. This points to the need to use RC or saturated analytic models for multiple period GRTs and not to rely either on RM-ANOVA analytic models with UN covariance or on an AIC or BIC favored model.

Future work in this area involves assessing the RM-ANOVA, RC, and saturated analytic models to other trials involving some component of group randomization, such as individually randomized treatment trials or stepped wedge group randomized designs [23, 39]. The performance of RC analytic models with these designs compared to RM-ANOVA has not been examined, according to a recent review of models for longitudinal GRTs [23]. Another area of potential study is comparing the RC, RM-ANOVA, and saturated analytic models for multiple period GRTs in the presence of an exponentially decaying cluster or individual autocorrelation. Recent papers in multiple-period GRTs have illuminated the need account for decaying autocorrelations in design and analysis [11, 22, 40]. The situation for RC models is more complicated, as the between-period ICC under the RC analytic model is a non-constant function of time.

## Conclusions

We found time × group RC and saturated analytic models using Kenward-Roger degrees of freedom maintained nominal type I error rate when applied to all data sets generated under a cohort and a cross-sectional parallel GRT design. We therefore recommend these analytic models for multiple-period parallel GRTs for both cross-sectional and cohort data, allowing an investigator to choose whether to model time as continuous or categorical. Analytic models specifying only group-level intercepts exhibited substantially inflated type I error rate unless the ICC was very low. This suggests time × group random effects are important to include in analytic models, as most investigators will not know in advance whether the time × group component of variance is zero.

## Supplementary information

**Additional file 1.** SAS code used to generate data and fit analytic models, as well as R code to combine SAS output and produce the figures.

**Additional file 2.** Type I error rate tables for analytic models.

## Data Availability

Simulation data can be recreated using the provided code.

## Abbreviations

GRT: Group randomized trial
RC: Random coefficients
RM-ANOVA: Repeated measures analysis of variance
UN: Unstructured
VC: Variance components
ICC: Intraclass correlation
CAC: Cluster autocorrelation
CS: Compound symmetry
IAC: Individual autocorrelation
BW: Between-within
