# Supplementary material for: Analysis of multiple-period group randomized trials: random coefficients model or repeated measures ANOVA?
Source: Trials. 2022 Dec 7;23:987. doi: 10.1186/s13063-022-06917-2 (PMC9727985; doi:10.1186/s13063-022-06917-2)
Supplement: Supplementary file 1 — Additional file 1. SAS code used to generate data and fit analytic models, as well as R code to combine SAS output and produce the figures. [file 13063_2022_6917_MOESM1_ESM.zip › R code for graphsR1.docx]

library(tidyverse)

library(kableExtra)

# rc, rmanova, sat, kr2 ddf

cross.ftests.dir <- "results/cross/ftests/3-rmanova-rc/"

cohort.ftests.dir <- "results/cohort/ftests/0-rmanova-rc-sat/"

# read in SAS results ----------------------------------------------------------

cross.ftests.fnames <- list.files(cross.ftests.dir)

cross.ftests.paths <- paste0(cross.ftests.dir,cross.ftests.fnames)

cross.ftests.list <- vector("list", length(cross.ftests.paths))

cohort.ftests.fnames <- list.files(cohort.ftests.dir)

cohort.ftests.paths <- paste0(cohort.ftests.dir,cohort.ftests.fnames)

cohort.ftests.list <- vector("list", length(cohort.ftests.paths))

# Combine lists ----------------------------------------------------------------

ftests.fnames <- c(cross.ftests.fnames, cohort.ftests.fnames)

ftests.paths <- c(cross.ftests.paths, cohort.ftests.paths)

ftests.list <- c(cross.ftests.list, cohort.ftests.list)

for(i in 1:length(ftests.list)){

i.split <- str_split(ftests.paths[i],"_",simplify = TRUE)

i.split.1 <- i.split[,1]

design <- NA

if(str_detect(i.split.1,"cohort")){

design <- "cohort"

} else {

design <- "cross"

}

dgm <- i.split[,4] # data generating model

type <- i.split[,6] # analytic model type

ranef <- i.split[,8]

var_g <- as.numeric(i.split[,12])

var_gt <- as.numeric(i.split[,14])

var_s <- as.numeric(i.split[16])

var_st <- as.numeric(i.split[18])

var_e <- as.numeric(i.split[,20])

icc <- NA

if(design == "cross"){

icc <- (var_g+var_gt)/(var_g+var_gt+var_e)

} else if(dgm == "rmatg"){

icc <- (var_g+var_gt)/(var_g+var_gt+var_s+var_e)

} else {

icc <- (var_g+var_gt)/(var_g+var_gt+var_s+var_st+var_e)

}

m <- as.numeric(i.split[,22])

tp <- as.numeric(i.split[,26])+1 # time periods

ddf <- str_replace(i.split[,28],pattern=fixed(".csv"),"")

ftests.list[[i]] <- read.csv(ftests.paths[i],stringsAsFactors = FALSE) %>%

mutate(design=design,

var_g = var_g,

var_gt = var_gt,

var_s = var_s,

var_st = var_st,

var_e = var_e,

icc = icc,

tp = tp,

m=m,

dgm = dgm,

type = type,

ranef = ranef,

pvalue = as.numeric(ProbF),

pvalue = ifelse(is.na(pvalue),0,pvalue),

fp = 1*(pvalue <= 0.05)) %>%

select(-ProbF)

}

df <- bind_rows(ftests.list)

cross.summ <- df %>%

filter(design == "cross") %>%

mutate(group = paste(dgm,type,ranef,icc,m,sep="_")) %>%

group_by(group) %>%

summarize(nfp = sum(fp), fpr = format(nfp/1000, digits=3,nsmall=3)) %>%

separate(group,sep="_",into=c("dgm","type","ranef","icc","m")) %>%

mutate(icc=round(as.numeric(icc),4)) %>%

mutate(ranef = ifelse(ranef == "g", "Group Intercept", "Time by Group")) %>%

mutate(data = "Cross-Sectional",

ICC = format(icc, digits = 4)) %>%

mutate(dgm = ifelse(dgm == "rc", "RC",dgm),

dgm = ifelse(dgm == "rma", "RM-ANOVA",dgm)) %>%

mutate(type = ifelse(type == "vc", "RM-ANOVA, VC", type),

type = ifelse(type == "un", "RM-ANOVA, UN", type),

type = ifelse(type == "rc", "RC", type),

type = ifelse(type == "sat", "Saturated", type)) %>%

mutate(fpr = as.numeric(fpr)) %>%

arrange(ranef, type) %>%

select(data,dgm,type,m,ranef,ICC,fpr)

cross.summ$ranef <- factor(cross.summ$ranef,

levels = c("Group Intercept", "Time by Group"))

cross.summ$type <- factor(cross.summ$type,

levels = c("RM-ANOVA, VC", "RM-ANOVA, UN", "RC","Saturated"))

# cross.summ$ICC <- fct_rev(factor(cross.summ$ICC))

cross.summ$ICC <- factor(cross.summ$ICC)

cross.summ$dgm <- factor(cross.summ$dgm,

levels = c("RM-ANOVA","RC"))

cohort.summ <- df %>%

filter(design == "cohort") %>%

mutate(group = paste(dgm,type,ranef,icc,m,sep="_")) %>%

group_by(group) %>%

summarize(nfp = sum(fp), fpr = format(nfp/1000, digits=3,nsmall=3)) %>%

separate(group,sep="_",into=c("dgm","type","ranef","icc","m")) %>%

mutate(icc=round(as.numeric(icc),4)) %>%

mutate(ranef = ifelse(ranef == "g", "Group Intercept", "Time by Group")) %>%

mutate(data = "Cohort",

ICC = format(icc, digits = 4)) %>%

mutate(dgm = ifelse(dgm == "rc", "RC",dgm),

dgm = ifelse(dgm %in% c("rma", "rmatg"), "RM-ANOVA",dgm)) %>%

mutate(type = ifelse(type == "vc", "RM-ANOVA, VC", type),

type = ifelse(type == "un", "RM-ANOVA, UN", type),

type = ifelse(type == "rc", "RC", type),

type = ifelse(type == "sat", "Saturated", type)) %>%

mutate(fpr = as.numeric(fpr)) %>%

arrange(ranef, type) %>%

select(data,dgm,type,m,ranef,ICC,fpr)

cohort.summ$ranef <- factor(cohort.summ$ranef,

levels = c("Group Intercept", "Time by Group"))

cohort.summ$type <- factor(cohort.summ$type,

levels = c("RM-ANOVA, VC", "RM-ANOVA, UN", "RC","Saturated"))

# cohort.summ$ICC <- fct_rev(factor(cohort.summ$ICC))

cohort.summ$ICC <- factor(cohort.summ$ICC)

cohort.summ$dgm <- factor(cohort.summ$dgm,

levels = c("RM-ANOVA","RC"))

both.summ <- rbind(cross.summ,cohort.summ)

g.plot <- both.summ %>%

filter(ranef == "Group Intercept") %>%

mutate(ranef = droplevels(ranef))

tg.plot <- both.summ %>%

filter(ranef == "Time by Group") %>%

mutate(ranef = droplevels(ranef))

axis.text.size <- 12

axis.title.size <- 14

strip.text.size <- 12

legend.text.size <- 12

legend.title.size <- 14

plot.title.size <- 14

g.plot.slides <- ggplot(g.plot, aes(x=ICC, y=fpr, group = type, color = type)) +

geom_point() +

geom_line() +

geom_hline(yintercept=0.05, color = "gray") +

facet_grid(m ~ dgm + data) +

ylim(0,0.9) +

labs(x = "Within-Period ICC", y = "Type I Error Rate", group = "",

color = "Analytic Model", title = "Group Intercept") +

# labs(x = "Within-Period ICC", y = "Type I Error Rate", group = "",

# color = "Analytic Model", subtitle = "Data Generation Mechanism", title = "Top") +

theme(axis.text = element_text(size=axis.text.size),

axis.title = element_text(size=axis.title.size),

axis.text.x = element_text(angle = -45, vjust = 0, hjust=0),

strip.text = element_text(size=strip.text.size),

legend.text = element_text(size=legend.text.size),

legend.title = element_text(size=legend.title.size),

plot.subtitle = element_text(size=plot.title.size,hjust = 0.5),

legend.position = "bottom") +

guides(color = guide_legend(title.position = "top",title.hjust=0.5))

g.plot.slide.name <- paste0(g.plot$ranef[1]," with KR2 ddf.png")

ggsave(g.plot.slide.name,g.plot.slides,width=9.5,height=6,dpi=800)

tg.plot.slides <- ggplot(tg.plot, aes(x=ICC, y=fpr, group = type, color = type)) +

geom_point() +

geom_line() +

geom_hline(yintercept=0.05, color = "gray") +

facet_grid(m ~ dgm + data) +

ylim(0,0.2) +

labs(x = "Within-Period ICC", y = "Type I Error Rate", group = "",

color = "Analytic Model", title = "Time by Group") +

# labs(x = "Within-Period ICC", y = "Type I Error Rate", group = "",

# color = "Analytic Model", subtitle = "Data Generation Mechanism", title = "Top") +

theme(axis.text = element_text(size=axis.text.size),

axis.title = element_text(size=axis.title.size),

axis.text.x = element_text(angle = -45, vjust = 0, hjust=0),

strip.text = element_text(size=strip.text.size),

legend.text = element_text(size=legend.text.size),

legend.title = element_text(size=legend.title.size),

plot.subtitle = element_text(size=plot.title.size,hjust = 0.5),

legend.position = "bottom") +

guides(color = guide_legend(title.position = "top",title.hjust=0.5))

tg.plot.slide.name <- paste0(tg.plot$ranef[1]," with KR2 ddf and Saturated.png")

ggsave(tg.plot.slide.name,tg.plot.slides,width=9.5,height=6,dpi=800)

options(knitr.kable.NA = '-')

g.tab <- g.plot %>%

select(-ranef) %>%

pivot_wider(names_from = c("dgm","data","ICC"), values_from = "fpr") %>%

select(m,type,

`RM-ANOVA_Cohort_0.001`,`RM-ANOVA_Cohort_0.010`,`RM-ANOVA_Cohort_0.100`,

`RM-ANOVA_Cross-Sectional_0.001`,`RM-ANOVA_Cross-Sectional_0.010`,`RM-ANOVA_Cross-Sectional_0.100`,

`RC_Cohort_0.001`,`RC_Cohort_0.010`,`RC_Cohort_0.100`,

`RC_Cross-Sectional_0.001`,`RC_Cross-Sectional_0.010`,`RC_Cross-Sectional_0.100`) %>%

arrange(m,type) %>%

mutate(m = ifelse(duplicated(m), " ", as.character(m)))

kable(g.tab,

col.names = c("M","Analytic Model",

rep(c("0.001","0.010","0.100"),4)),

caption="Group Intercept, KR ddf",

booktabs=TRUE,

linesep = c("","","\\addlinespace"),

format = "latex") %>%

kable_styling(latex_options = c("hold_position","scale_down")) %>%

add_header_above(c(" " = 2, "Cohort" = 3, "Cross-Sectional" = 3, "Cohort" = 3, "Cross-Sectional" = 3)) %>%

add_header_above(c(" " = 2, "RM-ANOVA" = 6, "RC" = 6))

options(knitr.kable.NA = '-')

tg.tab <- tg.plot %>%

select(-ranef) %>%

pivot_wider(names_from = c("dgm","data","ICC"), values_from = "fpr") %>%

select(m,type,

`RM-ANOVA_Cohort_0.001`,`RM-ANOVA_Cohort_0.010`,`RM-ANOVA_Cohort_0.100`,

`RM-ANOVA_Cross-Sectional_0.001`,`RM-ANOVA_Cross-Sectional_0.010`,`RM-ANOVA_Cross-Sectional_0.100`,

`RC_Cohort_0.001`,`RC_Cohort_0.010`,`RC_Cohort_0.100`,

`RC_Cross-Sectional_0.001`,`RC_Cross-Sectional_0.010`,`RC_Cross-Sectional_0.100`) %>%

arrange(m,type) %>%

mutate(m = ifelse(duplicated(m), " ", as.character(m)))

kable(tg.tab,

col.names = c("M","Analytic Model",

rep(c("0.001","0.010","0.100"),4)),

caption="Time x Group, KR ddf",

booktabs=TRUE,

linesep = c("","","","\\addlinespace"),

format = "latex") %>%

kable_styling(latex_options = c("hold_position","scale_down")) %>%

add_header_above(c(" " = 2, "Cohort" = 3, "Cross-Sectional" = 3, "Cohort" = 3, "Cross-Sectional" = 3)) %>%

add_header_above(c(" " = 2, "RM-ANOVA" = 6, "RC" = 6))
