# Supplementary material for: Analysis of multiple-period group randomized trials: random coefficients model or repeated measures ANOVA?
Source: Trials. 2022 Dec 7;23:987. doi: 10.1186/s13063-022-06917-2 (PMC9727985; doi:10.1186/s13063-022-06917-2)
Supplement: Supplementary file 1 — Additional file 1. SAS code used to generate data and fit analytic models, as well as R code to combine SAS output and produce the figures. [file 13063_2022_6917_MOESM1_ESM.zip › SAS code for analytic modelsR1.docx]

/*******************************************************************************/

/* Cross-Sectional Analytic Models */

/*******************************************************************************/

/*RM-ANOVA VC, int *************************************************************/

proc mixed data=XC method=reml nobound;

by sampID;

class cond group time;

model y = cond time cond*time/ddfm=kr2;

random int /subject=group(cond) type=vc G;

run;

/*******************************************************************************/

/*RM-ANOVA UN, int *************************************************************/

proc mixed data=XC method=reml nobound;

by sampID;

class cond group time;

model y = cond time cond*time/ddfm=kr2;

random int/subject=group(cond) type=un G;

run;

/*******************************************************************************/

/*RC, int **********************************************************************/

proc mixed data=XC method=reml nobound;

by sampID;

class cond group;

model y = cond time cond*time/ddfm=kr2;

random int/subject=group(cond) type=un G;

run;

/*******************************************************************************/

/*RM-ANOVA VC, int time ********************************************************/

proc mixed data=XC method=reml nobound;

by sampID;

class cond group time;

model y = cond time cond*time/ddfm=kr2;

random int time/subject=group(cond) type=vc G;

run;

/*******************************************************************************/

/*RM-ANOVA UN, int time individual *********************************************/

proc mixed data=XC method=reml;

by sampID;

class cond group time;

model y = cond time cond*time/ddfm=kr2;

random int time/subject=group(cond) type=un G;

run;

/*******************************************************************************/

/*RM-ANOVA UN, int time means **************************************************/

proc mixed data=XCmeans method=reml nobound;

by sampID;

class cond group time;

model y = cond time cond*time/ddfm=kr2;

repeated time/subject=group(cond) type=un R;

run;

/*******************************************************************************/

/*RC, int time *****************************************************************/

proc mixed data=XC method=reml nobound;

by sampID;

class cond group;

model y = cond time cond*time/ddfm=kr2;

random int time/subject=group(cond) type=un G;

run;

/*******************************************************************************/

/*SAT, individual **************************************************************/

proc mixed data=XC method=reml nobound;

by sampID;

class cond group person time;

model y=cond time cond*time/ddfm=kr2;

random time/subject=group(cond) type=un G;

repeated time/subject=person(group*cond) type=un(1) R;

run;

/*******************************************************************************/

/*SAT, mean ********************************************************************/

proc mixed data=XCmeans method=reml nobound;

by sampID;

class cond group time;

model y = cond time cond*time/ddfm=kr2;

repeated time/subject=group(cond) type=un R;

run;

/*******************************************************************************/

/*******************************************************************************/

/* Cohort Analytic Models */

/*******************************************************************************/

/*RM-ANOVA VC, int *************************************************************/

proc mixed data=CO method=reml nobound;

by sampID;

class cond group person time;

model y=cond time cond*time/ddfm=kr2;

random int/subject=group(cond) type=vc G;

repeated time/subject=person(group*cond) type=cs R;

run;

/*******************************************************************************/

/*RM-ANOVA UN, int *************************************************************/

proc mixed data=CO method=reml nobound;

by sampID;

class cond group person time;

model y=cond time cond*time/ddfm=kr2 s;

random int/subject=group(cond) type=vc G;

repeated time/type=un subject=person(group*cond) R;

run;

/*******************************************************************************/

/*RC, int **********************************************************************/

proc mixed data=CO method=reml nobound;

by sampID;

class cond group person;

model y=cond time cond*time/ddfm=kr2;

random int time/subject=group(cond) type=vc;

random int/subject=person(group*cond) type=un G;

run;

/*******************************************************************************/

/*RM-ANOVA VC, int time ********************************************************/

proc mixed data=CO method=reml nobound;

by sampID;

class cond group person time;

model y=cond time cond*time/ddfm=kr2;

random int time/subject=group(cond) type=vc G;

repeated time/subject=person(group*cond) type=cs R;

run;

/*******************************************************************************/

/*RM-ANOVA UN, int time ********************************************************/

proc mixed data=CO method=reml nobound;

by sampID;

class cond group person time;

model y=cond time cond*time/ddfm=kr2;

random int time/subject=group(cond) type=vc G;

repeated time/type=un subject=person(group*cond) R;

run;

/*******************************************************************************/

/*RC, int time *****************************************************************/

proc mixed data=CO method=reml;

by sampID;

class cond group person;

model y=cond time cond*time/ddfm=kr2;

random int time/subject=group(cond) type=un G;

random int time/subject=person(group*cond) type=un;

run;

/*******************************************************************************/

/*SAT, individual **************************************************************/

proc mixed data=CO method=reml nobound;

by sampID;

class cond group person time;

model y=cond time cond*time/ddfm=kr2;

random time/subject=group(cond) type=un G;

repeated time/subject=person(group*cond) type=un R;

run; /*******************************************************************************/

/*SAT, mean ********************************************************************/

proc mixed data=COmeans method=reml nobound;

by sampID;

class cond group time;

model y=cond time cond*time/ddfm=kr2;

repeated time / subject=group(cond) type=un;

run;

/*******************************************************************************/
