# Supplementary material for: Analysis of multiple-period group randomized trials: random coefficients model or repeated measures ANOVA?
Source: Trials. 2022 Dec 7;23:987. doi: 10.1186/s13063-022-06917-2 (PMC9727985; doi:10.1186/s13063-022-06917-2)
Supplement: Supplementary file 1 — Additional file 1. SAS code used to generate data and fit analytic models, as well as R code to combine SAS output and produce the figures. [file 13063_2022_6917_MOESM1_ESM.zip › SAS code for simulationsR1.docx]

| **Section** | **Page** |
| --- | --- |
| Generate cohort data | 1 |
| Analytic models for cohort RM-ANOVA data | 3 |
| Analytic models for cohort RC data | 8 |
| Generate cross-sectional data | 12 |
| Analytic models for cross-sectional RM-ANOVA data | 14 |
| Analytic models for cross-sectional RC data | 19 |

**Generate cohort data**

%let outdir = [enter path];

/*******************************************************************************/

%macro get_cohort_rmanova_data(nsamp, m, nper, tp, var_g, var_gt, var_s, var_res);

/*Generate, sort cohort RMANOVA data*/

/* Generate time by group random effects for group/cluster */

data CO1;

firstg = 1;

lastg = &m;

do sampID = 1 to &nsamp;

do cond = 0 to 1;

do group = firstg to lastg;

int_g = sqrt(&var_g)*rannor(0);

do time = 0 to &tp;

int_gt = sqrt(&var_gt)*rannor(0);

do person = 1 to &nper;

res = sqrt(&var_res)*rannor(0);

output;

end;

end;

end;

firstg = firstg + &m;

lastg = lastg + &m;

end;

end;

run;

/* Generate time-invariant random effects for member */

data CO2;

firstg = 1;

lastg = &m;

do sampID = 1 to &nsamp;

do cond = 0 to 1;

do group = firstg to lastg;

do person = 1 to &nper;

int_s = sqrt(&var_s)*rannor(0);

do time = 0 to &tp;

output;

end;

end;

end;

firstg = firstg + &m;

lastg = lastg + &m;

end;

end;

run;

proc sort data=CO2 out=CO2;

by sampID cond group time person;

run;

data COrmanova;

merge CO1 CO2;

y = int_g + int_gt + int_s + res;

keep sampID cond group time person y;

run;

proc sort data = COrmanova out = COrmanova;

by sampID cond group person time;

run;

proc means data = COrmanova noprint;

class sampID cond group time;

output out=COrmanova_means(where=(_type_ eq 15)) mean=;

var y;

run;

%mend;

/*******************************************************************************/

/*******************************************************************************/

%macro get_cohort_rancoef_data(nsamp, m, nper, tp, var_g, var_gt, var_s, var_st, var_res);

/*Generate, sort cohort RC data*/

data COrc;

keep sampID cond group time person y;

firstg = 1;

lastg = &m;

do sampID = 1 to &nsamp;

do cond = 0 to 1;

do group = firstg to lastg;

int_g = sqrt(&var_g)*rannor(0);

slope_g = sqrt(&var_gt)*rannor(0);

do person = 1 to &nper;

int_s = sqrt(&var_s)*rannor(0);

slope_s = sqrt(&var_st)*rannor(0);

do time = 0 to &tp;

res = sqrt(&var_res)*rannor(0);

y = int_g + slope_g*time + int_s + slope_s*time + res;

output;

end;

end;

end;

firstg = firstg + &m;

lastg = lastg + &m;

end;

end;

run;

proc sort data = COrc out = COrc;

by sampID cond group person time;

run;

proc means data = COrc noprint;

class sampID cond group time;

output out=COrc_means(where=(_type_ eq 15)) mean=;

var y;

run;

%mend;

/*******************************************************************************/

**Analytic models for cohort RM-ANOVA data**

/*******************************************************************************/

%macro run_cohort_rmanova_sim2(nsamp, m, nper, tp, var_g, var_gt, var_s, var_res, mymethod, myddf);

/*generate data set*/

ods graphics off;

ods exclude all;

/*RM-ANOVA VC, int *************************************************************/

proc mixed data = COrmanova method=&mymethod nobound;

by sampID;

class cond group person time;

model y=cond time cond*time/ddfm=&myddf;

random int/subject=group(cond) type=vc;

repeated time/subject=person(group*cond) type=cs;

ods output Tests3=Ftests ConvergenceStatus=ConvergeStatus FitStatistics=FitStats;

run;

proc export data = Ftests (where=(Effect eq "cond*time" or Effect eq "time*cond"))

outfile= "&outdir.\cohort\ftests\cohort_&mymethod._DGM_rmatg_MOD_vc_RANEF_g_NSAMP_&nsamp._VARG_&var_g._VARGT_&var_gt._VARS_&var_s._VARST_NA_VARE_&var_res._M_&m._NPER_&nper._TP_&tp._DDF_&myddf..csv"

dbms = CSV replace;

run;

proc export data = ConvergeStatus

outfile= "&outdir.\cohort\conv\cohort_&mymethod._DGM_rmatg_MOD_vc_RANEF_g_NSAMP_&nsamp._VARG_&var_g._VARGT_&var_gt._VARS_&var_s._VARST_NA_VARE_&var_res._M_&m._NPER_&nper._TP_&tp._DDF_&myddf..csv"

dbms = CSV replace;

run;

proc export data = FitStats

outfile= "&outdir.\cohort\fits\cohort_&mymethod._DGM_rmatg_MOD_vc_RANEF_g_NSAMP_&nsamp._VARG_&var_g._VARGT_&var_gt._VARS_&var_s._VARST_NA_VARE_&var_res._M_&m._NPER_&nper._TP_&tp._DDF_&myddf..csv"

dbms = CSV replace;

run;

/*RM-ANOVA UN, int *************************************************************/

proc mixed data = COrmanova method=&mymethod nobound;

by sampID;

class cond group person time;

model y=cond time cond*time/ddfm=&myddf;

random int/subject=group(cond) type=vc;

repeated time/subject=person(group*cond) type=un;

ods output Tests3=Ftests ConvergenceStatus=ConvergeStatus FitStatistics=FitStats;

run;

proc export data = Ftests (where=(Effect eq "cond*time" or Effect eq "time*cond"))

outfile= "&outdir.\cohort\ftests\cohort_&mymethod._DGM_rmatg_MOD_un_RANEF_g_NSAMP_&nsamp._VARG_&var_g._VARGT_&var_gt._VARS_&var_s._VARST_NA_VARE_&var_res._M_&m._NPER_&nper._TP_&tp._DDF_&myddf..csv"

dbms = CSV replace;

run;

proc export data = ConvergeStatus

outfile= "&outdir.\cohort\conv\cohort_&mymethod._DGM_rmatg_MOD_un_RANEF_g_NSAMP_&nsamp._VARG_&var_g._VARGT_&var_gt._VARS_&var_s._VARST_NA_VARE_&var_res._M_&m._NPER_&nper._TP_&tp._DDF_&myddf..csv"

dbms = CSV replace;

run;

proc export data = FitStats

outfile= "&outdir.\cohort\fits\cohort_&mymethod._DGM_rmatg_MOD_un_RANEF_g_NSAMP_&nsamp._VARG_&var_g._VARGT_&var_gt._VARS_&var_s._VARST_NA_VARE_&var_res._M_&m._NPER_&nper._TP_&tp._DDF_&myddf..csv"

dbms = CSV replace;

run;

/*RC, int **********************************************************************/

proc mixed data=COrmanova method=&mymethod nobound;

by sampID;

class cond group person;

model y=cond time cond*time/ddfm=&myddf;

random int/subject=group(cond) type=un;

random int/subject=person(group*cond) type=un;

ods output Tests3=Ftests ConvergenceStatus=ConvergeStatus FitStatistics=FitStats;

run;

proc export data = Ftests (where=(Effect eq "cond*time" or Effect eq "time*cond"))

outfile= "&outdir.\cohort\ftests\cohort_&mymethod._DGM_rmatg_MOD_rc_RANEF_g_NSAMP_&nsamp._VARG_&var_g._VARGT_&var_gt._VARS_&var_s._VARST_NA_VARE_&var_res._M_&m._NPER_&nper._TP_&tp._DDF_&myddf..csv"

dbms = CSV replace;

run;

proc export data = ConvergeStatus

outfile= "&outdir.\cohort\conv\cohort_&mymethod._DGM_rmatg_MOD_rc_RANEF_g_NSAMP_&nsamp._VARG_&var_g._VARGT_&var_gt._VARS_&var_s._VARST_NA_VARE_&var_res._M_&m._NPER_&nper._TP_&tp._DDF_&myddf..csv"

dbms = CSV replace;

run;

proc export data = FitStats

outfile= "&outdir.\cohort\fits\cohort_&mymethod._DGM_rmatg_MOD_rc_RANEF_g_NSAMP_&nsamp._VARG_&var_g._VARGT_&var_gt._VARS_&var_s._VARST_NA_VARE_&var_res._M_&m._NPER_&nper._TP_&tp._DDF_&myddf..csv"

dbms = CSV replace;

run;

/*RM-ANOVA VC, int time ********************************************************/

proc mixed data = cormanova method=&mymethod nobound;

by sampid;

class cond group person time;

model y=cond time cond*time/ddfm=&myddf;

random int time/subject=group(cond) type=vc;

repeated time/subject=person(group*cond) type=cs;

ods output Tests3=Ftests ConvergenceStatus=ConvergeStatus FitStatistics=FitStats;

run;

proc export data = Ftests (where=(effect eq "cond*time" or effect eq "time*cond"))

outfile= "&outdir.\cohort\ftests\cohort_&mymethod._DGM_rmatg_mod_vc_ranef_tg_nsamp_&nsamp._varg_&var_g._vargt_&var_gt._vars_&var_s._varst_na_vare_&var_res._m_&m._nper_&nper._tp_&tp._ddf_&myddf..csv"

dbms = csv replace;

run;

proc export data = ConvergeStatus

outfile= "&outdir.\cohort\conv\cohort_&mymethod._DGM_rmatg_mod_vc_ranef_tg_nsamp_&nsamp._varg_&var_g._vargt_&var_gt._vars_&var_s._varst_na_vare_&var_res._m_&m._nper_&nper._tp_&tp._ddf_&myddf..csv"

dbms = csv replace;

run;

proc export data = FitStats

outfile= "&outdir.\cohort\fits\cohort_&mymethod._DGM_rmatg_mod_vc_ranef_tg_nsamp_&nsamp._varg_&var_g._vargt_&var_gt._vars_&var_s._varst_na_vare_&var_res._m_&m._nper_&nper._tp_&tp._ddf_&myddf..csv"

dbms = csv replace;

run;

/*RM-ANOVA UN, int time ********************************************************/

proc mixed data = COrmanova method=&mymethod nobound;

by sampID;

class cond group person time;

model y=cond time cond*time/ddfm=&myddf;

random int time/subject=group(cond) type=vc;

repeated time/subject=person(group*cond) type=un;

ods output Tests3=Ftests ConvergenceStatus=ConvergeStatus FitStatistics=FitStats;

run;

proc export data = Ftests (where=(Effect eq "cond*time" or Effect eq "time*cond"))

outfile= "&outdir.\cohort\ftests\cohort_&mymethod._DGM_rmatg_MOD_un_RANEF_tg_NSAMP_&nsamp._VARG_&var_g._VARGT_&var_gt._VARS_&var_s._VARST_NA_VARE_&var_res._M_&m._NPER_&nper._TP_&tp._DDF_&myddf..csv"

dbms = CSV replace;

run;

proc export data = ConvergeStatus

outfile= "&outdir.\cohort\conv\cohort_&mymethod._DGM_rmatg_MOD_un_RANEF_tg_NSAMP_&nsamp._VARG_&var_g._VARGT_&var_gt._VARS_&var_s._VARST_NA_VARE_&var_res._M_&m._NPER_&nper._TP_&tp._DDF_&myddf..csv"

dbms = CSV replace;

run;

proc export data = FitStats

outfile= "&outdir.\cohort\fits\cohort_&mymethod._DGM_rmatg_MOD_un_RANEF_tg_NSAMP_&nsamp._VARG_&var_g._VARGT_&var_gt._VARS_&var_s._VARST_NA_VARE_&var_res._M_&m._NPER_&nper._TP_&tp._DDF_&myddf..csv"

dbms = CSV replace;

run;

/*RC, int time *****************************************************************/

proc mixed data=COrmanova method=&mymethod nobound;

by sampID;

class cond group person;

model y=cond time cond*time/ddfm=&myddf;

random int time/subject=group(cond) type=un;

random int time/subject=person(group*cond) type=un;

ods output Tests3=Ftests ConvergenceStatus=ConvergeStatus FitStatistics=FitStats;

run;

proc export data = Ftests (where=(Effect eq "cond*time" or Effect eq "time*cond"))

outfile= "&outdir.\cohort\ftests\cohort_&mymethod._DGM_rmatg_MOD_rc_RANEF_tg_NSAMP_&nsamp._VARG_&var_g._VARGT_&var_gt._VARS_&var_s._VARST_NA_VARE_&var_res._M_&m._NPER_&nper._TP_&tp._DDF_&myddf..csv"

dbms = CSV replace;

run;

proc export data = ConvergeStatus

outfile= "&outdir.\cohort\conv\cohort_&mymethod._DGM_rmatg_MOD_rc_RANEF_tg_NSAMP_&nsamp._VARG_&var_g._VARGT_&var_gt._VARS_&var_s._VARST_NA_VARE_&var_res._M_&m._NPER_&nper._TP_&tp._DDF_&myddf..csv"

dbms = CSV replace;

run;

proc export data = FitStats

outfile= "&outdir.\cohort\fits\cohort_&mymethod._DGM_rmatg_MOD_rc_RANEF_tg_NSAMP_&nsamp._VARG_&var_g._VARGT_&var_gt._VARS_&var_s._VARST_NA_VARE_&var_res._M_&m._NPER_&nper._TP_&tp._DDF_&myddf..csv"

dbms = CSV replace;

run;

/* sat *******************************************************************/

proc mixed data = COrmanova_means method=&mymethod nobound;

by sampID;

class cond group time;

model y=cond time cond*time/ddfm=&myddf;

repeated time / subject=group(cond) type=un;

ods output Tests3=Ftests ConvergenceStatus=ConvergeStatus FitStatistics=FitStats;

run;

proc export data = Ftests (where=(Effect eq "cond*time" or Effect eq "time*cond"))

outfile= "&outdir.\cohort\ftests\cohort_&mymethod._DGM_rmatg_MOD_sat_RANEF_tg_NSAMP_&nsamp._VARG_&var_g._VARGT_&var_gt._VARS_&var_s._VARST_NA_VARE_&var_res._M_&m._NPER_&nper._TP_&tp._DDF_&myddf..csv"

dbms = CSV replace;

run;

proc export data = ConvergeStatus

outfile= "&outdir.\cohort\conv\cohort_&mymethod._DGM_rmatg_MOD_sat_RANEF_tg_NSAMP_&nsamp._VARG_&var_g._VARGT_&var_gt._VARS_&var_s._VARST_NA_VARE_&var_res._M_&m._NPER_&nper._TP_&tp._DDF_&myddf..csv"

dbms = CSV replace;

run;

proc export data = FitStats

outfile= "&outdir.\cohort\fits\cohort_&mymethod._DGM_rmatg_MOD_sat_RANEF_tg_NSAMP_&nsamp._VARG_&var_g._VARGT_&var_gt._VARS_&var_s._VARST_NA_VARE_&var_res._M_&m._NPER_&nper._TP_&tp._DDF_&myddf..csv"

dbms = CSV replace;

run;

ods exclude none;

%mend;

/*******************************************************************************/

**Analytic models for cohort RC data**

/*******************************************************************************/

%macro run_cohort_rancoef_sim2(nsamp, m, nper, tp, var_g, var_gt, var_s, var_st, var_res, mymethod, myddf);

ods graphics off;

ods exclude all;

/*RM-ANOVA VC, int *************************************************************/

proc mixed data = COrc method=&mymethod nobound;

by sampID;

class cond group person time;

model y=cond time cond*time/ddfm=&myddf;

random int/subject=group(cond) type=vc;

repeated time/subject=person(group*cond) type=cs;

ods output Tests3=Ftests ConvergenceStatus=ConvergeStatus FitStatistics=FitStats;

run;

proc export data = Ftests (where=(Effect eq "cond*time" or Effect eq "time*cond"))

outfile= "&outdir.\cohort\ftests\cohort_&mymethod._DGM_rctg_MOD_vc_RANEF_g_NSAMP_&nsamp._VARG_&var_g._VARGT_&var_gt._VARS_&var_s._VARST_&var_st._VARE_&var_res._M_&m._NPER_&nper._TP_&tp._DDF_&myddf..csv"

dbms = CSV replace;

run;

proc export data = ConvergeStatus

outfile= "&outdir.\cohort\conv\cohort_&mymethod._DGM_rctg_MOD_vc_RANEF_g_NSAMP_&nsamp._VARG_&var_g._VARGT_&var_gt._VARS_&var_s._VARST_&var_st._VARE_&var_res._M_&m._NPER_&nper._TP_&tp._DDF_&myddf..csv"

dbms = CSV replace;

run;

proc export data = FitStats

outfile= "&outdir.\cohort\fits\cohort_&mymethod._DGM_rctg_MOD_vc_RANEF_g_NSAMP_&nsamp._VARG_&var_g._VARGT_&var_gt._VARS_&var_s._VARST_&var_st._VARE_&var_res._M_&m._NPER_&nper._TP_&tp._DDF_&myddf..csv"

dbms = CSV replace;

run;

/*RM-ANOVA UN, int *************************************************************/

proc mixed data = COrc method=&mymethod nobound;

by sampID;

class cond group person time;

model y=cond time cond*time/ddfm=&myddf;

random int/subject=group(cond) type=vc;

repeated time/subject=person(group*cond) type=un;

ods output Tests3=Ftests ConvergenceStatus=ConvergeStatus FitStatistics=FitStats;

run;

proc export data = Ftests (where=(Effect eq "cond*time" or Effect eq "time*cond"))

outfile= "&outdir.\cohort\ftests\cohort_&mymethod._DGM_rctg_MOD_un_RANEF_g_NSAMP_&nsamp._VARG_&var_g._VARGT_&var_gt._VARS_&var_s._VARST_&var_st._VARE_&var_res._M_&m._NPER_&nper._TP_&tp._DDF_&myddf..csv"

dbms = CSV replace;

run;

proc export data = ConvergeStatus

outfile= "&outdir.\cohort\conv\cohort_&mymethod._DGM_rctg_MOD_un_RANEF_g_NSAMP_&nsamp._VARG_&var_g._VARGT_&var_gt._VARS_&var_s._VARST_&var_st._VARE_&var_res._M_&m._NPER_&nper._TP_&tp._DDF_&myddf..csv"

dbms = CSV replace;

run;

proc export data = FitStats

outfile= "&outdir.\cohort\fits\cohort_&mymethod._DGM_rctg_MOD_un_RANEF_g_NSAMP_&nsamp._VARG_&var_g._VARGT_&var_gt._VARS_&var_s._VARST_&var_st._VARE_&var_res._M_&m._NPER_&nper._TP_&tp._DDF_&myddf..csv"

dbms = CSV replace;

run;

/*RC, int **********************************************************************/

proc mixed data=COrc method=&mymethod nobound;

by sampID;

class cond group person;

model y=cond time cond*time/ddfm=&myddf;

random int/subject=group(cond) type=un;

random int time/subject=person(group*cond) type=un;

ods output Tests3=Ftests ConvergenceStatus=ConvergeStatus FitStatistics=FitStats;

run;

proc export data = Ftests (where=(Effect eq "cond*time" or Effect eq "time*cond"))

outfile= "&outdir.\cohort\ftests\cohort_&mymethod._DGM_rctg_MOD_rc_RANEF_g_NSAMP_&nsamp._VARG_&var_g._VARGT_&var_gt._VARS_&var_s._VARST_&var_st._VARE_&var_res._M_&m._NPER_&nper._TP_&tp._DDF_&myddf..csv"

dbms = CSV replace;

run;

proc export data = ConvergeStatus

outfile= "&outdir.\cohort\conv\cohort_&mymethod._DGM_rctg_MOD_rc_RANEF_g_NSAMP_&nsamp._VARG_&var_g._VARGT_&var_gt._VARS_&var_s._VARST_&var_st._VARE_&var_res._M_&m._NPER_&nper._TP_&tp._DDF_&myddf..csv"

dbms = CSV replace;

run;

proc export data = FitStats

outfile= "&outdir.\cohort\fits\cohort_&mymethod._DGM_rctg_MOD_rc_RANEF_g_NSAMP_&nsamp._VARG_&var_g._VARGT_&var_gt._VARS_&var_s._VARST_&var_st._VARE_&var_res._M_&m._NPER_&nper._TP_&tp._DDF_&myddf..csv"

dbms = CSV replace;

run;

/*RM-ANOVA VC, int time ********************************************************/

proc mixed data = COrc method=&mymethod nobound;

by sampID;

class cond group person time;

model y=cond time cond*time/ddfm=&myddf;

random int time/subject=group(cond) type=vc;

repeated time/subject=person(group*cond) type=cs;

ods output Tests3=Ftests ConvergenceStatus=ConvergeStatus FitStatistics=FitStats;

run;

proc export data = Ftests (where=(Effect eq "cond*time" or Effect eq "time*cond"))

outfile= "&outdir.\cohort\ftests\cohort_&mymethod._DGM_rctg_MOD_vc_RANEF_tg_NSAMP_&nsamp._VARG_&var_g._VARGT_&var_gt._VARS_&var_s._VARST_&var_st._VARE_&var_res._M_&m._NPER_&nper._TP_&tp._DDF_&myddf..csv"

dbms = CSV replace;

run;

proc export data = ConvergeStatus

outfile= "&outdir.\cohort\conv\cohort_&mymethod._DGM_rctg_MOD_vc_RANEF_tg_NSAMP_&nsamp._VARG_&var_g._VARGT_&var_gt._VARS_&var_s._VARST_&var_st._VARE_&var_res._M_&m._NPER_&nper._TP_&tp._DDF_&myddf..csv"

dbms = CSV replace;

run;

proc export data = FitStats

outfile= "&outdir.\cohort\fits\cohort_&mymethod._DGM_rctg_MOD_vc_RANEF_tg_NSAMP_&nsamp._VARG_&var_g._VARGT_&var_gt._VARS_&var_s._VARST_&var_st._VARE_&var_res._M_&m._NPER_&nper._TP_&tp._DDF_&myddf..csv"

dbms = CSV replace;

run;

/*RM-ANOVA UN, int time ********************************************************/

proc mixed data = COrc method=&mymethod nobound;

by sampID;

class cond group person time;

model y=cond time cond*time/ddfm=&myddf;

random int time/subject=group(cond) type=vc;

repeated time/subject=person(group*cond) type=un;

ods output Tests3=Ftests ConvergenceStatus=ConvergeStatus FitStatistics=FitStats;

run;

proc export data = Ftests (where=(Effect eq "cond*time" or Effect eq "time*cond"))

outfile= "&outdir.\cohort\ftests\cohort_&mymethod._DGM_rctg_MOD_un_RANEF_tg_NSAMP_&nsamp._VARG_&var_g._VARGT_&var_gt._VARS_&var_s._VARST_&var_st._VARE_&var_res._M_&m._NPER_&nper._TP_&tp._DDF_&myddf..csv"

dbms = CSV replace;

run;

proc export data = ConvergeStatus

outfile= "&outdir.\cohort\conv\cohort_&mymethod._DGM_rctg_MOD_un_RANEF_tg_NSAMP_&nsamp._VARG_&var_g._VARGT_&var_gt._VARS_&var_s._VARST_&var_st._VARE_&var_res._M_&m._NPER_&nper._TP_&tp._DDF_&myddf..csv"

dbms = CSV replace;

run;

proc export data = FitStats

outfile= "&outdir.\cohort\fits\cohort_&mymethod._DGM_rctg_MOD_un_RANEF_tg_NSAMP_&nsamp._VARG_&var_g._VARGT_&var_gt._VARS_&var_s._VARST_&var_st._VARE_&var_res._M_&m._NPER_&nper._TP_&tp._DDF_&myddf..csv"

dbms = CSV replace;

run;

/*RC, int time *****************************************************************/

proc mixed data=COrc method=&mymethod nobound;

by sampID;

class cond group person;

model y=cond time cond*time/ddfm=&myddf;

random int time/subject=group(cond) type=un;

random int time/subject=person(group*cond) type=un;

ods output Tests3=Ftests ConvergenceStatus=ConvergeStatus FitStatistics=FitStats;

run;

proc export data = Ftests (where=(Effect eq "cond*time" or Effect eq "time*cond"))

outfile= "&outdir.\cohort\ftests\cohort_&mymethod._DGM_rctg_MOD_rc_RANEF_tg_NSAMP_&nsamp._VARG_&var_g._VARGT_&var_gt._VARS_&var_s._VARST_&var_st._VARE_&var_res._M_&m._NPER_&nper._TP_&tp._DDF_&myddf..csv"

dbms = CSV replace;

run;

proc export data = ConvergeStatus

outfile= "&outdir.\cohort\conv\cohort_&mymethod._DGM_rctg_MOD_rc_RANEF_tg_NSAMP_&nsamp._VARG_&var_g._VARGT_&var_gt._VARS_&var_s._VARST_&var_st._VARE_&var_res._M_&m._NPER_&nper._TP_&tp._DDF_&myddf..csv"

dbms = CSV replace;

run;

proc export data = FitStats

outfile= "&outdir.\cohort\fits\cohort_&mymethod._DGM_rctg_MOD_rc_RANEF_tg_NSAMP_&nsamp._VARG_&var_g._VARGT_&var_gt._VARS_&var_s._VARST_&var_st._VARE_&var_res._M_&m._NPER_&nper._TP_&tp._DDF_&myddf..csv"

dbms = CSV replace;

run;

/* sat *******************************************************************/

proc mixed data = COrc_means method=&mymethod nobound;

by sampID;

class cond group time;

model y=cond time cond*time/ddfm=&myddf;

repeated time / subject=group(cond) type=un;

ods output Tests3=Ftests ConvergenceStatus=ConvergeStatus FitStatistics=FitStats;

run;

proc export data = Ftests (where=(Effect eq "cond*time" or Effect eq "time*cond"))

outfile= "&outdir.\cohort\ftests\cohort_&mymethod._DGM_rctg_MOD_sat_RANEF_tg_NSAMP_&nsamp._VARG_&var_g._VARGT_&var_gt._VARS_&var_s._VARST_&var_st._VARE_&var_res._M_&m._NPER_&nper._TP_&tp._DDF_&myddf..csv"

dbms = CSV replace;

run;

proc export data = ConvergeStatus

outfile= "&outdir.\cohort\conv\cohort_&mymethod._DGM_rctg_MOD_sat_RANEF_tg_NSAMP_&nsamp._VARG_&var_g._VARGT_&var_gt._VARS_&var_s._VARST_&var_st._VARE_&var_res._M_&m._NPER_&nper._TP_&tp._DDF_&myddf..csv"

dbms = CSV replace;

run;

proc export data = FitStats

outfile= "&outdir.\cohort\fits\cohort_&mymethod._DGM_rctg_MOD_sat_RANEF_tg_NSAMP_&nsamp._VARG_&var_g._VARGT_&var_gt._VARS_&var_s._VARST_&var_st._VARE_&var_res._M_&m._NPER_&nper._TP_&tp._DDF_&myddf..csv"

dbms = CSV replace;

run;

ods exclude none;

%mend;

/*******************************************************************************/

**Generate cross-sectional data**

%let outdir = [enter path];

/*******************************************************************************/

%macro get_cross_rmanova_data(nsamp, m, nper, tp, var_g, var_gt, var_res);

/*Generate, sort, summarize, and export cross-sectional RMANOVA data*/

data XCrmanova;

firstg = 1;

lastg = &m;

do sampID = 1 to &nsamp;

do cond = 0 to 1;

do group = firstg to lastg;

int_g = sqrt(&var_g)*rannor(0);

do time = 0 to &tp;

int_gt = sqrt(&var_gt)*rannor(0);

do person = 1 to &nper;

res = sqrt(&var_res)*rannor(0);

y = int_g + int_gt + res;

output;

end;

end;

end;

firstg = firstg + &m;

lastg = lastg + &m;

end;

end;

proc sort data = XCrmanova out = XCrmanova;

by sampID cond group time person;

run;

proc means data = XCrmanova noprint;

class sampID cond group time;

output out=XCrmanova_means(where=(_type_ eq 15)) mean=;

var y;

run;

%mend;

/*******************************************************************************/

/*******************************************************************************/

%macro get_cross_rancoef_data(nsamp, m, nper, tp, var_g, var_gt, var_res);

/*Generate, sort, summarize, and export cross-sectional RC data*/

data XCrc;

firstg = 1;

lastg = &m;

do sampID = 1 to &nsamp;

do cond = 0 to 1;

do group = firstg to lastg;

int = sqrt(&var_g)*rannor(0);

slope = sqrt(&var_gt)*rannor(0);

do time = 0 to &tp;

do person = 1 to &nper;

res = sqrt(&var_res)*rannor(0);

y = int + slope*time + res;

output;

end;

end;

end;

firstg = firstg + &m;

lastg = lastg + &m;

end;

end;

proc sort data = XCrc out = XCrc;

by sampID cond group time person;

run;

proc means data = XCrc noprint;

class sampID cond group time;

output out=XCrc_means(where=(_type_ eq 15)) mean=;

var y;

run;

%mend;

/*******************************************************************************/

**Analytic models for cross-sectional RM-ANOVA data**

/*******************************************************************************/

%macro run_cross_rmanova_sim2(nsamp, m, nper, tp, var_g, var_gt, var_res, mymethod, myddf);

/*generate data set*/

ods graphics off;

ods exclude all;

/*RM-ANOVA VC, int *************************************************************/

proc mixed data = XCrmanova method=&mymethod nobound;

by sampID;

class cond group time;

model y=cond time cond*time/ddfm=&myddf;

random int/subject=group(cond) type=vc;

ods output Tests3=Ftests ConvergenceStatus=ConvergeStatus FitStatistics=FitStats;

run;

proc export data = Ftests (where=(Effect eq "cond*time" or Effect eq "time*cond"))

outfile= "&outdir.\cross\ftests\cross_&mymethod._DGM_rma_MOD_vc_RANEF_g_NSAMP_&nsamp._VARG_&var_g._VARGT_&var_gt._VARS_NA_VARST_NA_VARE_&var_res._M_&m._NPER_&nper._TP_&tp._DDF_&myddf..csv"

dbms = CSV replace;

run;

proc export data = ConvergeStatus

outfile= "&outdir.\cross\conv\cross_&mymethod._DGM_rma_MOD_vc_RANEF_g_NSAMP_&nsamp._VARG_&var_g._VARGT_&var_gt._VARS_NA_VARST_NA_VARE_&var_res._M_&m._NPER_&nper._TP_&tp._DDF_&myddf..csv"

dbms = CSV replace;

run;

proc export data = FitStats

outfile= "&outdir.\cross\fits\cross_&mymethod._DGM_rma_MOD_vc_RANEF_g_NSAMP_&nsamp._VARG_&var_g._VARGT_&var_gt._VARS_NA_VARST_NA_VARE_&var_res._M_&m._NPER_&nper._TP_&tp._DDF_&myddf..csv"

dbms = CSV replace;

run;

/*RM-ANOVA UN, int *************************************************************/

proc mixed data = XCrmanova method=&mymethod nobound;

by sampID;

class cond group time;

model y = cond time cond*time/ddfm=&myddf;

random int/subject=group(cond) type=un;

ods output Tests3=Ftests ConvergenceStatus=ConvergeStatus FitStatistics=FitStats;

run;

proc export data = Ftests (where=(Effect eq "cond*time" or Effect eq "time*cond"))

outfile= "&outdir.\cross\ftests\cross_&mymethod._DGM_rma_MOD_un_RANEF_g_NSAMP_&nsamp._VARG_&var_g._VARGT_&var_gt._VARS_NA_VARST_NA_VARE_&var_res._M_&m._NPER_&nper._TP_&tp._DDF_&myddf..csv"

dbms = CSV replace;

run;

proc export data = ConvergeStatus

outfile= "&outdir.\cross\conv\cross_&mymethod._DGM_rma_MOD_un_RANEF_g_NSAMP_&nsamp._VARG_&var_g._VARGT_&var_gt._VARS_NA_VARST_NA_VARE_&var_res._M_&m._NPER_&nper._TP_&tp._DDF_&myddf..csv"

dbms = CSV replace;

run;

proc export data = FitStats

outfile= "&outdir.\cross\fits\cross_&mymethod._DGM_rma_MOD_un_RANEF_g_NSAMP_&nsamp._VARG_&var_g._VARGT_&var_gt._VARS_NA_VARST_NA_VARE_&var_res._M_&m._NPER_&nper._TP_&tp._DDF_&myddf..csv"

dbms = CSV replace;

run;

/*RC, int **********************************************************************/

proc mixed data=XCrmanova method=&mymethod nobound;

by sampID;

class cond group;

model y = cond time cond*time/ddfm=&myddf;

random int/subject=group(cond) type=un;

ods output Tests3=Ftests ConvergenceStatus=ConvergeStatus FitStatistics=FitStats;

run;

proc export data = Ftests (where=(Effect eq "cond*time" or Effect eq "time*cond"))

outfile= "&outdir.\cross\ftests\cross_&mymethod._DGM_rma_MOD_rc_RANEF_g_NSAMP_&nsamp._VARG_&var_g._VARGT_&var_gt._VARS_NA_VARST_NA_VARE_&var_res._M_&m._NPER_&nper._TP_&tp._DDF_&myddf..csv"

dbms = CSV replace;

run;

proc export data = ConvergeStatus

outfile= "&outdir.\cross\conv\cross_&mymethod._DGM_rma_MOD_rc_RANEF_g_NSAMP_&nsamp._VARG_&var_g._VARGT_&var_gt._VARS_NA_VARST_NA_VARE_&var_res._M_&m._NPER_&nper._TP_&tp._DDF_&myddf..csv"

dbms = CSV replace;

run;

proc export data = FitStats

outfile= "&outdir.\cross\fits\cross_&mymethod._DGM_rma_MOD_rc_RANEF_g_NSAMP_&nsamp._VARG_&var_g._VARGT_&var_gt._VARS_NA_VARST_NA_VARE_&var_res._M_&m._NPER_&nper._TP_&tp._DDF_&myddf..csv"

dbms = CSV replace;

run;

/*RM-ANOVA VC, int time ********************************************************/

proc mixed data = XCrmanova method=&mymethod nobound;

by sampID;

class cond group time;

model y = cond time cond*time/ddfm=&myddf;

random int time/subject=group(cond) type=vc;

ods output Tests3=Ftests ConvergenceStatus=ConvergeStatus FitStatistics=FitStats;

run;

proc export data = Ftests (where=(Effect eq "cond*time" or Effect eq "time*cond"))

outfile= "&outdir.\cross\ftests\cross_&mymethod._DGM_rma_MOD_vc_RANEF_tg_NSAMP_&nsamp._VARG_&var_g._VARGT_&var_gt._VARS_NA_VARST_NA_VARE_&var_res._M_&m._NPER_&nper._TP_&tp._DDF_&myddf..csv"

dbms = CSV replace;

run;

proc export data = ConvergeStatus

outfile= "&outdir.\cross\conv\cross_&mymethod._DGM_rma_MOD_vc_RANEF_tg_NSAMP_&nsamp._VARG_&var_g._VARGT_&var_gt._VARS_NA_VARST_NA_VARE_&var_res._M_&m._NPER_&nper._TP_&tp._DDF_&myddf..csv"

dbms = CSV replace;

run;

proc export data = FitStats

outfile= "&outdir.\cross\fits\cross_&mymethod._DGM_rma_MOD_vc_RANEF_tg_NSAMP_&nsamp._VARG_&var_g._VARGT_&var_gt._VARS_NA_VARST_NA_VARE_&var_res._M_&m._NPER_&nper._TP_&tp._DDF_&myddf..csv"

dbms = CSV replace;

run;

/*RM-ANOVA UN, int time ********************************************************/

proc mixed data = XCrmanova_means method=&mymethod nobound;

by sampID;

class cond group time;

model y = cond time cond*time/ddfm=&myddf;

repeated time/subject=group(cond) type=un;

ods output Tests3=Ftests ConvergenceStatus=ConvergeStatus FitStatistics=FitStats;

run;

proc export data = Ftests (where=(Effect eq "cond*time" or Effect eq "time*cond"))

outfile= "&outdir.\cross\ftests\cross_&mymethod._DGM_rma_MOD_un_RANEF_tg_NSAMP_&nsamp._VARG_&var_g._VARGT_&var_gt._VARS_NA_VARST_NA_VARE_&var_res._M_&m._NPER_&nper._TP_&tp._DDF_&myddf..csv"

dbms = CSV replace;

run;

proc export data = ConvergeStatus

outfile= "&outdir.\cross\conv\cross_&mymethod._DGM_rma_MOD_un_RANEF_tg_NSAMP_&nsamp._VARG_&var_g._VARGT_&var_gt._VARS_NA_VARST_NA_VARE_&var_res._M_&m._NPER_&nper._TP_&tp._DDF_&myddf..csv"

dbms = CSV replace;

run;

proc export data = FitStats

outfile= "&outdir.\cross\fits\cross_&mymethod._DGM_rma_MOD_un_RANEF_tg_NSAMP_&nsamp._VARG_&var_g._VARGT_&var_gt._VARS_NA_VARST_NA_VARE_&var_res._M_&m._NPER_&nper._TP_&tp._DDF_&myddf..csv"

dbms = CSV replace;

run;

/*RC, int time *****************************************************************/

proc mixed data=XCrmanova method=&mymethod nobound;

by sampID;

class cond group;

model y = cond time cond*time/ddfm=&myddf;

random int time/subject=group(cond) type=un;

ods output Tests3=Ftests ConvergenceStatus=ConvergeStatus FitStatistics=FitStats;

run;

proc export data = Ftests (where=(Effect eq "cond*time" or Effect eq "time*cond"))

outfile= "&outdir.\cross\ftests\cross_&mymethod._DGM_rma_MOD_rc_RANEF_tg_NSAMP_&nsamp._VARG_&var_g._VARGT_&var_gt._VARS_NA_VARST_NA_VARE_&var_res._M_&m._NPER_&nper._TP_&tp._DDF_&myddf..csv"

dbms = CSV replace;

run;

proc export data = ConvergeStatus

outfile= "&outdir.\cross\conv\cross_&mymethod._DGM_rma_MOD_rc_RANEF_tg_NSAMP_&nsamp._VARG_&var_g._VARGT_&var_gt._VARS_NA_VARST_NA_VARE_&var_res._M_&m._NPER_&nper._TP_&tp._DDF_&myddf..csv"

dbms = CSV replace;

run;

proc export data = FitStats

outfile= "&outdir.\cross\fits\cross_&mymethod._DGM_rma_MOD_rc_RANEF_tg_NSAMP_&nsamp._VARG_&var_g._VARGT_&var_gt._VARS_NA_VARST_NA_VARE_&var_res._M_&m._NPER_&nper._TP_&tp._DDF_&myddf..csv"

dbms = CSV replace;

run;

/*sat **************************************************************************/

proc mixed data=XCrmanova_means method=&mymethod nobound;

by sampID;

class cond group time;

model y = cond time cond*time/ddfm=&myddf;

repeated time/subject=group(cond) type=un;

ods output Tests3=Ftests ConvergenceStatus=ConvergeStatus FitStatistics=FitStats;

run;

proc export data = Ftests (where=(Effect eq "cond*time" or Effect eq "time*cond"))

outfile= "&outdir.\cross\ftests\cross_&mymethod._DGM_rma_MOD_sat_RANEF_tg_NSAMP_&nsamp._VARG_&var_g._VARGT_&var_gt._VARS_NA_VARST_NA_VARE_&var_res._M_&m._NPER_&nper._TP_&tp._DDF_&myddf..csv"

dbms = CSV replace;

run;

proc export data = ConvergeStatus

outfile= "&outdir.\cross\conv\cross_&mymethod._DGM_rma_MOD_sat_RANEF_tg_NSAMP_&nsamp._VARG_&var_g._VARGT_&var_gt._VARS_NA_VARST_NA_VARE_&var_res._M_&m._NPER_&nper._TP_&tp._DDF_&myddf..csv"

dbms = CSV replace;

run;

proc export data = FitStats

outfile= "&outdir.\cross\fits\cross_&mymethod._DGM_rma_MOD_sat_RANEF_tg_NSAMP_&nsamp._VARG_&var_g._VARGT_&var_gt._VARS_NA_VARST_NA_VARE_&var_res._M_&m._NPER_&nper._TP_&tp._DDF_&myddf..csv"

dbms = CSV replace;

run;

ods exclude none;

%mend;

/*******************************************************************************/

**Analytic models for cross-sectional RC data**

/*******************************************************************************/

%macro run_cross_rancoef_sim2(nsamp, m, nper, tp, var_g, var_gt, var_res, mymethod, myddf);

/*generate data set*/

ods graphics off;

ods exclude all;

/*RM-ANOVA VC, int *************************************************************/

proc mixed data = XCrc method=&mymethod nobound;

by sampID;

class cond group time;

model y=cond time cond*time/ddfm=&myddf;

random int/subject=group(cond) type=vc;

ods output Tests3=Ftests ConvergenceStatus=ConvergeStatus FitStatistics=FitStats;

run;

proc export data = Ftests (where=(Effect eq "cond*time" or Effect eq "time*cond"))

outfile= "&outdir.\cross\ftests\cross_&mymethod._DGM_rc_MOD_vc_RANEF_g_NSAMP_&nsamp._VARG_&var_g._VARGT_&var_gt._VARS_NA_VARST_NA_VARE_&var_res._M_&m._NPER_&nper._TP_&tp._DDF_&myddf..csv"

dbms = CSV replace;

run;

proc export data = ConvergeStatus

outfile= "&outdir.\cross\conv\cross_&mymethod._DGM_rc_MOD_vc_RANEF_g_NSAMP_&nsamp._VARG_&var_g._VARGT_&var_gt._VARS_NA_VARST_NA_VARE_&var_res._M_&m._NPER_&nper._TP_&tp._DDF_&myddf..csv"

dbms = CSV replace;

run;

proc export data = FitStats

outfile= "&outdir.\cross\fits\cross_&mymethod._DGM_rc_MOD_vc_RANEF_g_NSAMP_&nsamp._VARG_&var_g._VARGT_&var_gt._VARS_NA_VARST_NA_VARE_&var_res._M_&m._NPER_&nper._TP_&tp._DDF_&myddf..csv"

dbms = CSV replace;

run;

/*RM-ANOVA UN, int *************************************************************/

proc mixed data = XCrc method=&mymethod nobound;

by sampID;

class cond group time;

model y = cond time cond*time/ddfm=&myddf;

random int/subject=group(cond) type=un;

ods output Tests3=Ftests ConvergenceStatus=ConvergeStatus FitStatistics=FitStats;

run;

proc export data = Ftests (where=(Effect eq "cond*time" or Effect eq "time*cond"))

outfile= "&outdir.\cross\ftests\cross_&mymethod._DGM_rc_MOD_un_RANEF_g_NSAMP_&nsamp._VARG_&var_g._VARGT_&var_gt._VARS_NA_VARST_NA_VARE_&var_res._M_&m._NPER_&nper._TP_&tp._DDF_&myddf..csv"

dbms = CSV replace;

run;

proc export data = ConvergeStatus

outfile= "&outdir.\cross\conv\cross_&mymethod._DGM_rc_MOD_un_RANEF_g_NSAMP_&nsamp._VARG_&var_g._VARGT_&var_gt._VARS_NA_VARST_NA_VARE_&var_res._M_&m._NPER_&nper._TP_&tp._DDF_&myddf..csv"

dbms = CSV replace;

run;

proc export data = FitStats

outfile= "&outdir.\cross\fits\cross_&mymethod._DGM_rc_MOD_un_RANEF_g_NSAMP_&nsamp._VARG_&var_g._VARGT_&var_gt._VARS_NA_VARST_NA_VARE_&var_res._M_&m._NPER_&nper._TP_&tp._DDF_&myddf..csv"

dbms = CSV replace;

run;

/*RC, int **********************************************************************/

proc mixed data=XCrc method=&mymethod nobound;

by sampID;

class cond group;

model y = cond time cond*time/ddfm=&myddf;

random int/subject=group(cond) type=un;

ods output Tests3=Ftests ConvergenceStatus=ConvergeStatus FitStatistics=FitStats;

run;

proc export data = Ftests (where=(Effect eq "cond*time" or Effect eq "time*cond"))

outfile= "&outdir.\cross\ftests\cross_&mymethod._DGM_rc_MOD_rc_RANEF_g_NSAMP_&nsamp._VARG_&var_g._VARGT_&var_gt._VARS_NA_VARST_NA_VARE_&var_res._M_&m._NPER_&nper._TP_&tp._DDF_&myddf..csv"

dbms = CSV replace;

run;

proc export data = ConvergeStatus

outfile= "&outdir.\cross\conv\cross_&mymethod._DGM_rc_MOD_rc_RANEF_g_NSAMP_&nsamp._VARG_&var_g._VARGT_&var_gt._VARS_NA_VARST_NA_VARE_&var_res._M_&m._NPER_&nper._TP_&tp._DDF_&myddf..csv"

dbms = CSV replace;

run;

proc export data = FitStats

outfile= "&outdir.\cross\fits\cross_&mymethod._DGM_rc_MOD_rc_RANEF_g_NSAMP_&nsamp._VARG_&var_g._VARGT_&var_gt._VARS_NA_VARST_NA_VARE_&var_res._M_&m._NPER_&nper._TP_&tp._DDF_&myddf..csv"

dbms = CSV replace;

run;

/*RM-ANOVA VC, int time ********************************************************/

proc mixed data = XCrc method=&mymethod nobound;

by sampID;

class cond group time;

model y = cond time cond*time/ddfm=&myddf;

random int time/subject=group(cond) type=vc;

ods output Tests3=Ftests ConvergenceStatus=ConvergeStatus FitStatistics=FitStats;

run;

proc export data = Ftests (where=(Effect eq "cond*time" or Effect eq "time*cond"))

outfile= "&outdir.\cross\ftests\cross_&mymethod._DGM_rc_MOD_vc_RANEF_tg_NSAMP_&nsamp._VARG_&var_g._VARGT_&var_gt._VARS_NA_VARST_NA_VARE_&var_res._M_&m._NPER_&nper._TP_&tp._DDF_&myddf..csv"

dbms = CSV replace;

run;

proc export data = ConvergeStatus

outfile= "&outdir.\cross\conv\cross_&mymethod._DGM_rc_MOD_vc_RANEF_tg_NSAMP_&nsamp._VARG_&var_g._VARGT_&var_gt._VARS_NA_VARST_NA_VARE_&var_res._M_&m._NPER_&nper._TP_&tp._DDF_&myddf..csv"

dbms = CSV replace;

run;

proc export data = FitStats

outfile= "&outdir.\cross\fits\cross_&mymethod._DGM_rc_MOD_vc_RANEF_tg_NSAMP_&nsamp._VARG_&var_g._VARGT_&var_gt._VARS_NA_VARST_NA_VARE_&var_res._M_&m._NPER_&nper._TP_&tp._DDF_&myddf..csv"

dbms = CSV replace;

run;

/*RM-ANOVA UN, int time ********************************************************/

proc mixed data = XCrc_means method=&mymethod nobound;

by sampID;

class cond group time;

model y = cond time cond*time/ddfm=&myddf;

repeated time/subject=group(cond) type=un;

ods output Tests3=Ftests ConvergenceStatus=ConvergeStatus FitStatistics=FitStats;

run;

proc export data = Ftests (where=(Effect eq "cond*time" or Effect eq "time*cond"))

outfile= "&outdir.\cross\ftests\cross_&mymethod._DGM_rc_MOD_un_RANEF_tg_NSAMP_&nsamp._VARG_&var_g._VARGT_&var_gt._VARS_NA_VARST_NA_VARE_&var_res._M_&m._NPER_&nper._TP_&tp._DDF_&myddf..csv"

dbms = CSV replace;

run;

proc export data = ConvergeStatus

outfile= "&outdir.\cross\conv\cross_&mymethod._DGM_rc_MOD_un_RANEF_tg_NSAMP_&nsamp._VARG_&var_g._VARGT_&var_gt._VARS_NA_VARST_NA_VARE_&var_res._M_&m._NPER_&nper._TP_&tp._DDF_&myddf..csv"

dbms = CSV replace;

run;

proc export data = FitStats

outfile= "&outdir.\cross\fits\cross_&mymethod._DGM_rc_MOD_un_RANEF_tg_NSAMP_&nsamp._VARG_&var_g._VARGT_&var_gt._VARS_NA_VARST_NA_VARE_&var_res._M_&m._NPER_&nper._TP_&tp._DDF_&myddf..csv"

dbms = CSV replace;

run;

/*RC, int time *****************************************************************/

proc mixed data=XCrc method=&mymethod nobound;

by sampID;

class cond group;

model y = cond time cond*time/ddfm=&myddf;

random int time/subject=group(cond) type=un;

ods output Tests3=Ftests ConvergenceStatus=ConvergeStatus FitStatistics=FitStats;

run;

proc export data = Ftests (where=(Effect eq "cond*time" or Effect eq "time*cond"))

outfile= "&outdir.\cross\ftests\cross_&mymethod._DGM_rc_MOD_rc_RANEF_tg_NSAMP_&nsamp._VARG_&var_g._VARGT_&var_gt._VARS_NA_VARST_NA_VARE_&var_res._M_&m._NPER_&nper._TP_&tp._DDF_&myddf..csv"

dbms = CSV replace;

run;

proc export data = ConvergeStatus

outfile= "&outdir.\cross\conv\cross_&mymethod._DGM_rc_MOD_rc_RANEF_tg_NSAMP_&nsamp._VARG_&var_g._VARGT_&var_gt._VARS_NA_VARST_NA_VARE_&var_res._M_&m._NPER_&nper._TP_&tp._DDF_&myddf..csv"

dbms = CSV replace;

run;

proc export data = FitStats

outfile= "&outdir.\cross\fits\cross_&mymethod._DGM_rc_MOD_rc_RANEF_tg_NSAMP_&nsamp._VARG_&var_g._VARGT_&var_gt._VARS_NA_VARST_NA_VARE_&var_res._M_&m._NPER_&nper._TP_&tp._DDF_&myddf..csv"

dbms = CSV replace;

run;

/*sat **************************************************************************/

proc mixed data=XCrc_means method=&mymethod nobound;

by sampID;

class cond group time;

model y = cond time cond*time/ddfm=&myddf;

repeated time/subject=group(cond) type=un;

ods output Tests3=Ftests ConvergenceStatus=ConvergeStatus FitStatistics=FitStats;

run;

proc export data = Ftests (where=(Effect eq "cond*time" or Effect eq "time*cond"))

outfile= "&outdir.\cross\ftests\cross_&mymethod._DGM_rc_MOD_sat_RANEF_tg_NSAMP_&nsamp._VARG_&var_g._VARGT_&var_gt._VARS_NA_VARST_NA_VARE_&var_res._M_&m._NPER_&nper._TP_&tp._DDF_&myddf..csv"

dbms = CSV replace;

run;

proc export data = ConvergeStatus

outfile= "&outdir.\cross\conv\cross_&mymethod._DGM_rc_MOD_sat_RANEF_tg_NSAMP_&nsamp._VARG_&var_g._VARGT_&var_gt._VARS_NA_VARST_NA_VARE_&var_res._M_&m._NPER_&nper._TP_&tp._DDF_&myddf..csv"

dbms = CSV replace;

run;

proc export data = FitStats

outfile= "&outdir.\cross\fits\cross_&mymethod._DGM_rc_MOD_sat_RANEF_tg_NSAMP_&nsamp._VARG_&var_g._VARGT_&var_gt._VARS_NA_VARST_NA_VARE_&var_res._M_&m._NPER_&nper._TP_&tp._DDF_&myddf..csv"

dbms = CSV replace;

run;

ods exclude none;

%mend;

/*******************************************************************************/
