# Supplementary material for: Analysis of multiple-period group randomized trials: random coefficients model or repeated measures ANOVA?
Source: Trials. 2022 Dec 7;23:987. doi: 10.1186/s13063-022-06917-2 (PMC9727985; doi:10.1186/s13063-022-06917-2)
Supplement: Supplementary file 2 — Additional file 2. Type I error rate tables for analytic models. [file 13063_2022_6917_MOESM2_ESM.pdf]

1 Time by Group Analytic Models

Table 1: Time x Group, KR ddf

		RM-ANOVA						RC					
		Cohort			Cross-Sectional			Cohort			Cross-Sectional		
M	Analytic Model	0.001	0.010	0.100	0.001	0.010	0.100	0.001	0.010	0.100	0.001	0.010	0.100
10	RM-ANOVA, VC	0.040	0.057	0.047	0.055	0.058	0.050	0.067	0.102	0.133	0.049	0.064	0.127
	RM-ANOVA, UN	0.039	0.057	0.046	0.061	0.058	0.050	0.052	0.154	0.157	0.054	0.051	0.054
	RC	0.051	0.051	0.050	0.040	0.046	0.043	0.037	0.048	0.050	0.049	0.049	0.056
	Saturated	0.045	0.047	0.048	0.061	0.058	0.043	0.045	0.047	0.036	0.054	0.051	0.054
20	RM-ANOVA, VC	0.052	0.048	0.058	0.035	0.048	0.047	0.087	0.109	0.135	0.052	0.059	0.101
	RM-ANOVA, UN	0.055	0.049	0.058	0.044	0.048	0.046	0.064	0.151	0.159	0.050	0.038	0.047
	RC	0.052	0.048	0.052	0.042	0.052	0.045	0.060	0.050	0.051	0.061	0.050	0.039
	Saturated	0.058	0.052	0.055	0.044	0.048	0.046	0.053	0.041	0.050	0.050	0.038	0.047
40	RM-ANOVA, VC	0.064	0.042	0.057	0.052	0.058	0.047	0.069	0.094	0.124	0.047	0.057	0.115
	RM-ANOVA, UN	0.065	0.043	0.057	0.064	0.059	0.049	0.057	0.140	0.149	0.041	0.042	0.047
	RC	0.043	0.057	0.066	0.043	0.054	0.052	0.047	0.043	0.047	0.035	0.038	0.050
	Saturated	0.070	0.043	0.060	0.064	0.059	0.049	0.043	0.057	0.059	0.041	0.042	0.047

Table 2: Time x Group, BW ddf

		RM-ANOVA						RC					
		Cohort			Cross-Sectional			Cohort			Cross-Sectional		
M	Analytic Model	0.001	0.010	0.100	0.001	0.010	0.100	0.001	0.010	0.100	0.001	0.010	0.100
10	RM-ANOVA, VC	0.042	0.043	0.056	0.055	0.058	0.050	0.067	0.102	0.134	0.049	0.065	0.127
	RM-ANOVA, UN	0.048	0.046	0.056	0.141	0.131	0.134	0.054	0.162	0.160	0.133	0.141	0.142
	RC	0.055	0.042	0.045	0.040	0.046	0.043	0.037	0.048	0.050	0.049	0.049	0.056
	Saturated	0.138	0.144	0.140	0.141	0.131	0.134	0.126	0.127	0.113	0.133	0.141	0.142
20	RM-ANOVA, VC	0.053	0.050	0.049	0.035	0.047	0.046	0.086	0.109	0.134	0.052	0.059	0.100
	RM-ANOVA, UN	0.054	0.048	0.049	0.069	0.082	0.087	0.064	0.152	0.159	0.080	0.062	0.078
	RC	0.047	0.048	0.052	0.042	0.052	0.045	0.060	0.050	0.051	0.061	0.050	0.039
	Saturated	0.088	0.084	0.094	0.069	0.082	0.087	0.086	0.085	0.077	0.080	0.062	0.078
40	RM-ANOVA, VC	0.044	0.042	0.041	0.052	0.056	0.047	0.068	0.094	0.124	0.047	0.057	0.115
	RM-ANOVA, UN	0.044	0.041	0.042	0.081	0.074	0.064	0.057	0.141	0.149	0.056	0.056	0.062
	RC	0.054	0.047	0.049	0.043	0.054	0.052	0.047	0.043	0.047	0.035	0.038	0.050
	Saturated	0.061	0.060	0.062	0.081	0.074	0.064	0.054	0.063	0.075	0.056	0.056	0.062

2 Group Intercept Analytic Models

Table 3: Group Intercept, KR ddf

		RM-ANOVA						RC					
		Cohort			Cross-Sectional			Cohort			Cross-Sectional		
M	Analytic Model	0.001	0.010	0.100	0.001	0.010	0.100	0.001	0.010	0.100	0.001	0.010	0.100
10	RM-ANOVA, VC	0.061	0.225	0.845	0.047	0.103	0.536	0.101	0.307	0.676	0.054	0.179	0.601
	RM-ANOVA, UN	0.061	0.218	0.849	0.047	0.103	0.536	0.059	0.209	0.556	0.054	0.179	0.601
	RC	0.059	0.129	0.444	0.047	0.064	0.266	0.087	0.282	0.648	0.073	0.260	0.683
20	RM-ANOVA, VC	0.064	0.217	0.834	0.033	0.093	0.551	0.105	0.309	0.696	0.063	0.162	0.556
	RM-ANOVA, UN	0.064	0.218	0.832	0.033	0.093	0.551	0.069	0.198	0.567	0.063	0.162	0.556
	RC	0.050	0.125	0.446	0.045	0.073	0.247	0.092	0.282	0.674	0.078	0.254	0.643
40	RM-ANOVA, VC	0.080	0.228	0.856	0.061	0.105	0.531	0.092	0.309	0.681	0.056	0.164	0.577
	RM-ANOVA, UN	0.079	0.227	0.857	0.061	0.105	0.531	0.061	0.184	0.576	0.056	0.164	0.577
	RC	0.044	0.142	0.459	0.040	0.075	0.270	0.076	0.275	0.642	0.056	0.241	0.672

Table 4: Group Intercept, BW ddf

		RM-ANOVA						RC					
		Cohort			Cross-Sectional			Cohort			Cross-Sectional		
M	Analytic Model	0.001	0.010	0.100	0.001	0.010	0.100	0.001	0.010	0.100	0.001	0.010	0.100
10	RM-ANOVA, VC	0.033	0.075	0.514	0.040	0.093	0.503	0.088	0.298	0.670	0.046	0.167	0.582
	RM-ANOVA, UN	0.036	0.073	0.516	0.040	0.093	0.503	0.051	0.194	0.548	0.046	0.167	0.582
	RC	0.042	0.053	0.230	0.031	0.047	0.231	0.061	0.261	0.630	0.050	0.233	0.666
20	RM-ANOVA, VC	0.048	0.077	0.545	0.031	0.089	0.538	0.103	0.302	0.694	0.057	0.152	0.553
	RM-ANOVA, UN	0.048	0.074	0.547	0.031	0.089	0.538	0.068	0.188	0.558	0.057	0.152	0.553
	RC	0.042	0.066	0.252	0.035	0.064	0.235	0.082	0.268	0.661	0.070	0.239	0.633
40	RM-ANOVA, VC	0.044	0.087	0.511	0.059	0.101	0.525	0.087	0.301	0.680	0.054	0.158	0.575
	RM-ANOVA, UN	0.045	0.090	0.512	0.059	0.101	0.525	0.055	0.179	0.571	0.054	0.158	0.575
	RC	0.055	0.062	0.242	0.038	0.070	0.264	0.073	0.270	0.637	0.050	0.233	0.664

3 Data Generation Mechanism with no Time x Group Random Effect

Table 5: Cross-Sectional DGM with only Group Intercept variation and no Time x Group variation

		Group-Intercept			Time x Group		
Analytic Model	M	0.001	0.010	0.100	0.001	0.010	0.100
RC	10	0.066	0.041	0.049	0.060	0.034	0.043
	20	0.047	0.040	0.051	0.038	0.036	0.047
	40	0.045	0.050	0.059	0.046	0.050	0.058
RM-ANOVA with UN	10	0.058	0.047	0.057	0.054	0.040	0.049
	20	0.051	0.045	0.056	0.056	0.044	0.055
	40	0.053	0.060	0.060	0.049	0.062	0.054
RM-ANOVA with VC	10	0.058	0.047	0.057	0.060	0.037	0.049
	20	0.051	0.045	0.056	0.052	0.045	0.053
	40	0.053	0.060	0.060	0.049	0.062	0.061
Saturated	10	-	-	-	0.054	0.040	0.049
	20	-	-	-	0.056	0.044	0.055
	40	-	-	-	0.049	0.062	0.054

Table 6: Cohort

		RM-ANOVA				RC			
		Group Intercept		Time by Group		Group Intercept		Time by Group	
Analytic Model	M	0.010	0.100	0.010	0.100	0.010	0.100	0.010	0.100
RM-ANOVA, VC	10	0.059	0.048	0.064	0.051	0.059	0.058	0.062	0.069
	20	0.053	0.056	0.055	0.06	0.068	0.072	0.069	0.074
RM-ANOVA, UN	10	0.058	0.045	0.065	0.05	0.036	0.049	0.033	0.046
	20	0.054	0.058	0.056	0.058	0.058	0.047	0.05	0.047
RC	10	0.063	0.046	0.067	0.05	0.038	0.056	0.043	0.053
	20	0.053	0.052	0.052	0.052	0.052	0.047	0.051	0.045
Saturated	10	-	-	0.053	0.049	-	-	0.042	0.048
	20	-	-	0.062	0.061	-	-	0.051	0.045
